# Nationwide surveys of awareness of tuberculosis in India uncover a gender gap in tuberculosis awareness

**DOI:** 10.1038/s43856-024-00592-x

**Published:** 2024-08-23

**Authors:** Ranganath Thimmanahalli Sobagaiah, Nitu Kumari, Divya Bharathi Gattam, Mohammed Shoyaib Khazi

**Affiliations:** 1 Bangalore Medical College and Research Institute, Bengaluru, India; 2 World College of Medical Sciences and Research, Jhajjar, India; 3 Kempegowda Institute Medical Sciences, Bengaluru, India; 4 All India Institute of Medical Sciences, Mangalagiri, India

**Keywords:** Epidemiology, Tuberculosis

## Abstract

**Background:**

Tuberculosis remains a major challenge in India, with an estimated 2.69 million cases each year. Although men are more affected than women, gender differences and related factors affect awareness of tuberculosis and thus impact tuberculosis diagnosis and access to treatment. Understanding the gender-specific needs and complexities when diagnosing and treating tuberculosis is essential to manage cases in India.

**Methods:**

We undertook a comparative study using data from three National Family and Health Surveys (NFHS), specifically NFHS-3, NFHS-4 and NFHS-5. We investigated the prevalence and gender disparity in awareness about tuberculosis, and associated factors, using regression analysis.

**Results:**

Most men and women surveyed are between the ages of 15 and 19. Across the surveys, the proportion of men and women who are unaware of spreading of tuberculosis decreases from 44.9% during NFHS 3 to 29.6% during NFHS 5. However, the prevalence ratio of men to women with no knowledge about modes of transmission of Tuberculosis increases from 0.92 during NFHS 3 to 0.98 during NFHS 5. Higher odds with younger age (NFHS 5, aOR: 1.07 (1.01–1.13)) and rural residency (NFHS 5, aOR: 1.12 (1.06–1.18)), and lower odds with unmarried marital status (NFHS 5, aOR: 0.92 (0.86–0.98)) are noteworthy associations. Women and men have differences in knowledge.

**Conclusions:**

Gender disparity associated with awareness about tuberculosis in India is observed across all three nationwide surveys. Being aged fifteen to nineteen years and residing in rural area are risk factors. Being unmarried is a protective factor for women, but not for men.

## Plain Language Summary

Lack of awareness of the spread of tuberculosis may be an important factor contributing to the current burden of disease. We used datasets from three rounds of the National Family Health Survey conducted in India to determine the proportion of men and women who knew how tuberculosis spreads. Using a predictive model, we showed that misconceptions are more common among both men and women. For women, younger age and living in rural areas were risk factors for lack of awareness, which was not the case for men. Such differences may represent a barrier to reducing the burden of disease. These findings can be used to develop gender-specific, comprehensive people awareness programs to raise awareness about tuberculosis.

## Abstract (web summary)

Sobagaiah et al. investigate the gender gap in awareness about how tuberculosis is transmitted 2 across three national family health surveys in India. Regression modelling shows variation in 3 awareness between men and women at a national level.

## Introduction

Tuberculosis (TB) is a disease vastly influenced and prevented by the social factors in the community. Lack of knowledge regarding the disease can contribute to underuse of medical services, delay in diagnosis and poor treatment adherence in people living with tuberculosis. Enhancing the dissemination of information on tuberculosis to increase the public awareness and health promotion is crucial to achieve the global targets for reduction in disease burden of tuberculosis. Studies have revealed that irrespective of a general overview about the disease, there is a breach in knowledge regarding the transmission, diagnosis, management, and its prevention. Also, poor knowledge or comprehension of tuberculosis disease and its treatment frequently contributes to non-adherence to therapy^1–3^.

Currently in India, the National Tuberculosis Elimination Programme (NTEP) with the development of National Strategic Plan 2017–25 is an ambitious attempt by the Government to eliminate tuberculosis by 2025^4^. Despite being a preventable and curable disease, tuberculosis is the most infectious killer disease attributing to almost 10 million cases per year globally, out of which 1.9 million cases are from India^5,6^. Numerous guidelines and tools have been released and made accessible by the Ministry of Health and Family Welfare (MoHFW) to tackle the problem of tuberculosis. The policies have been constantly updated after gathering considerable implementation-related learnings and the expansion of programme activities is still happening^7^.

Lack of knowledge about TB is a continuing problem and pose a risk for its prevention and care in China^8^. Similar situation can also be expected in India. In addition, because of the lack of knowledge about the disease and fear of being ostracized, persons with TB often hide their symptoms and fail to receive appropriate treatment which is a stumbling block in the prevention and care of the disease^9^.

The trend analysis of National Family Health Survey (NFHS)^10^ aids to give us key themes to improve the National Tuberculosis Elimination Programme’s (NTEP) coverage, quality, equity, efficiency, and effectiveness.

Internationally, in countries with high disease burden of tuberculosis, the routine diagnosis of tuberculosis, treatment compliance and health seeking habits are observed to be affected by gender and their knowledge and perception towards the disease^11,12^. The overall misconceptions about the transmission of TB ranges from 43–68 percent of women and 35–66 percent of men in all subgroups of background characteristics^13,14^.

With the disease burden of 1,933,381 cases from India in 2021^6^, out of which 6% were children aged 0 to 14 years, 58% were men and 36% were women, it becomes even more crucial to address the gap in awareness of transmission of TB among the two genders. Therefore, the Central TB Division formed the National Framework for Gender Responsive Approach to TB in India guidelines which reports that gender differences and inequalities play a crucial role in how people access and receive healthcare due to TB^15^. Gender is an important variable in the incidence, exposure, risks, health seeking behaviour and in treatment outcomes of tuberculosis.

Globally, studies have also shown that men may repress their illnesses knowingly or unknowingly in an effort to avoid being perceived as weak or feminine, or as a form of compensation. They achieve this, among other things, by believing that they are physically superior to women. They ignore disease as they work to fulfil their obligations to support and uplift their families, something many people are finding harder and harder to accomplish^7^. Men perceive control as a fundamental component of acceptable manhood and efforts to obtain it have also led men to put their health on the back burner, men were afraid of being perceived as being less than men^11,16^.

In countries like Malawi, role constructions as primary material providers for their immediate family along with the opportunity costs of acknowledging illness seem important barriers to care-seeking. Upon that, Men’s sense of adequacy as providers was influenced by limited employment opportunities and small incomes. It has been suggested that there is a need to address harmful masculinity and promote gender equality to support interventions for TB and chronic cough^16^.

In India, men are more affected with TB compared to women, but women are at a higher risk of manifesting the disease easily due to undernutrition mainly because of social norms which prevent prioritizing of their nutrition, health, and well-being. Whereas men are at the risk of developing TB due to their employment like mining and construction industries^15^.

Moreover, the factors affecting the health seeking due to gender remains the same as that found globally and access to services is greatly impacted by gender disparities that affect care-seeking, as well as health system variables such access restrictions, a lower index of suspicion of TB in women, and the provision of insufficient information to care-seekers^17^.

Across the globe, there has been a trend that the female participation in the surveys exceeded male participation in TB related surveys^18^. During NFHS less number of men were interviewed when compared to women. Hence, gender-specific factors in tuberculosis prevention and treatment can have a wide range such as differences in care-seeking behavior, diagnostic challenges, risk factors, disease burden of HIV and tuberculosis coinfection, and delayed treatment. Addressing these factors is crucial for achieving equity in tuberculosis care and reducing the burden of the disease among both men and women^19^. Another important factor that can be considered is the sex assortativity among the contacts of the existing patients that might have contributed to sex disparities in disease burden of tuberculosis among adults^20^. According to the NFHS-5 data, although, gender influence in knowledge and perception towards the disease affects the tuberculosis management and care, the extent of the influence is not explicitly explored in India.

This study observes the trends in gender influence in awareness of transmission of tuberculosis at national level to understand the factors that affect this. Noteworthy variation in awareness regarding transmission of tuberculosis is observed among men and women at the national level. On exploring the factors that influence TB awareness, interesting results are obtained which have major implications for TB prevention and care initiatives such as the NTEP in India. The most important factors among women are socioeconomic status, rural residence, age, and education. Our results suggest empowering women and promoting the education of mothers could improve TB awareness, a goal of the TB prevention and care program in India.

## Methods

### Study design

It is a cross-sectional study that compares three complex sample surveys of nationally representative population.

### Data sources

Datasets of Demographic Health Survey (DHS) which is also known as National Family Health Survey (NFHS) in India. After permission we obtained the recoded datasets for all three NFHS from DHS. Individual Recode file that contains the data on all the women and Mens Recode file that contains data on all men interviewed during NFHS were used in data analysis. These files shall be referred as Womens dataset and Mens dataset in the article.

### Setting

For NFHS 3 conducted during 2005 to 2006, the survey included participants from 29 states. For NFHS 4 conducted during 2015 to 2016 and NFHS 5 conducted during 2019 to 2021, the survey included participants from all states and union territories.

### Sample characteristics

For NFHS surveys, the multistage cluster sampling is adopted along with population proportion to sampling technique.

### Participants

NFHS-3 and NFHS-4 adopted different sample designs for data collection. NFHS-3 used a two-stage approach for rural areas and a three-stage approach for urban areas. In rural areas, the first stage involved selecting villages as Primary Sampling Units (PSUs) using probability proportional to population size (PPS), and in the second stage, households were systematically chosen within each village. In urban areas, three stages were used, with the selection of wards, Census Enumeration Blocks (CEBs), and households^21^. NFHS-4 employed a stratified two-stage sample design with the 2011 census serving as the sampling frame. In rural areas, PSUs (villages) were selected using PPS, and the strata were defined based on the number of households and the percentage of the population belonging to scheduled castes and tribes. In urban areas, CEBs were selected using PPS, considering the SC/ST population percentage. Complete household mapping was conducted in selected PSUs, which were segmented into clusters. Random sampling was used to choose clusters, and within each selected cluster, 22 households were randomly selected in the second stage of data collection. This design resulted in NFHS-4 clusters being either complete PSUs or segments of PSUs^22^. NFHS-5 used the same sample design as that of NFHS-4^21^. From each household one woman from the eligible age group was selected randomly for interview. However for men, during NFHS 3, only those men were interviewed who were usual residents of the sample household or visitors who stayed in the sample household the night before the survey^21^. However, during NFHS 4 and NFHS 5, only men who were selected only in the subsample of households selected for the state module^22,23^. In addition to the above, during NFHS 3, the union territories were not considered. Moreover, Telangana was formed in June 2014. Therefore, it is not available as a separate state in NFHS 3. Similarly, Ladakh as a union territory was formed in October 2019. Hence it is not available as a separate state or union territory in NFHS 4.

A total of 74369 cases in Mens dataset and 124385 cases in Womens dataset during NFHS 3, 112122 cases in Mens dataset and 169686 cases in Womens dataset during NFHS 4 and 101839 cases in Mens dataset and 724115 cases in Womens dataset during NFHS 5 were available. Inclusion criteria for analysis for our research was, first: age group of the respondent between 15 to 45 years of age, and second was “Yes” as response to the question: Ever heard about tuberculosis. Detailed inclusion criteria are given in Supplementary Figs. 1 and 2.

### Variables

#### Dependent

We created the variable on awareness about tuberculosis based on the respondent’s response as “Yes” or “No” to the following questions that were asked during NFHS survey:

Q1: Tuberculosis spread by: Air when coughing or sneezing.

Q2: Tuberculosis spread by: Sharing utensils.

Q3: Tuberculosis spread by: Touching a person with tuberculosis.

Q4: Tuberculosis spread by: Food.

Q5: Tuberculosis spread by: Sexual contact.

Q6: Tuberculosis spread by: Mosquito bites.

Based on the responses, we derived four categories in the dependent variable which are as follows: Category 1: Knowledge without misconceptions: if the response was “Yes” to Q1 and “No” to all other questions. Category 2: Knowledge with misconceptions: if the response was “Yes” to Q1 and “Yes” to any other questions from Q2 to Q6. Category 3: No knowledge without misconceptions: if the response was “No” to all questions from Q1 to Q6. Category 4: No knowledge with misconceptions: if the response was “No” to Q1 and “Yes” to any other questions from Q2 to Q6. For data representation and analysis, Category 3 and Category 4 were added and was considered as single category. Category 1 was used as reference for regression analysis. The categorization in the dependent variable was made based on previous study^24^.

### Variables

#### Independent

Based on the review of literature^15^, we selected the following variables for the regression model.

### Age in five-year groups

The current age of the respondent was divided into groups of five years each. The participants from all surveys selected in the study were belonging to the age group of fifteen to forty-five years of age. Age group of 45 to 49 years was used as reference category.

### Type of place of residence

It is where the respondent was interviewed as either urban or rural which was created based on whether the cluster or sample point number is defined as urban or rural and urban area was considered as a reference category.

### State

Region in which the respondent was interviewed. During NFHS 3, only twenty-nine states were included. However, during NFHS 4 and NFHS 5, states along with Union Territories were also included in the survey. Kerala state was taken as a reference category.

### Highest education level

This is a standardized variable providing level of education in the following categories: No education, Primary, Secondary, and Higher which was used as reference category.

### Wealth Index

The wealth index is a composite measure of a household’s cumulative living standard. The wealth index is calculated using easy-to-collect data on a household’s ownership of selected assets, such as televisions and bicycles; materials used for housing construction; and types of water access and sanitation facilities. Richest category was used for reference in regression analysis.

### Current marital status

It is the current marital status of the respondent. The original variable in the dataset was recoded to form three categories as the distribution of data among various categories in the original variable was skewed. The recoded variable had three categories: “Never married”, “Married” and “Others” which was used as reference category.

### Response to the question

Tuberculosis can be cured: The response had three categories: “No”, “Yes” and “Don’t know”. The response “Yes” was taken as reference category.

### Response to the question

Keep secret if family member gets tuberculosis: The response had three categories: “No”, “Yes, remain a secret” and “Don’t know/Not sure/It depends”. Response “No” was taken as reference category.

### Frequency of reading newspaper or magazine

The response had four categories: “Not at all”, “Less than once a week”, “at least once a week” and “almost every day”.

### Frequency of listening to radio

The response was had four categories like those of frequency of reading newspaper or magazine.

### Frequency of watching television

The response was had four categories like those of frequency of reading newspaper or magazine. Reference category for frequency of reading newspaper or magazine, listening to radio and watching television was “at least once a week” for regression analysis.

### Bias

During NFHS 3, the Men’s Questionnaire was employed to interview men aged 15–54 who were usual residents of the sample household or visitors who stayed in the sample household the night before the survey^21^. However, during NFHS 4 and NFHS 5, the Men’s Questionnaire was administered only in the subsample of households selected for the state module^14,21^. Hence, the number of cases in the Mens dataset are less in number when compared to those in Womens dataset. Moreover, those who were not interviewed may have contributed notably to the results of our study.

### Study size

64,212 cases from Mens dataset and 109,032 cases from Womens dataset file for NFHS 3, 91,293 cases from Mens dataset and 61,8274 cases from Womens dataset for NFHS 4 and 85,751 cases from Mens dataset and 671,750 cases from Womens dataset for NFHS 5 were included in the study for further analysis.

### Ethical considerations

Our study used secondary data for analysis from the datasets provided by the Demographic Health Surveys Program (DHS). We applied for access, and this was granted based on us providing information about our planned use. All the datasets provided were re coded and already anonymized to completely protect the privacy of the survey participants. Informed consent was obtained from the participant or guardian (for children) before the interview for all surveys by DHS^25^. We did not obtain approval from institutional review boards as the data we were using was deidentified and recoded, that has already been reviewed for privacy and ethical concerns before by DHS. Moreover, this data is available public domain in form of datasets and national and state level reports. The authors were not allowed to share the datasets with each other. Hence all authors have obtained authorization to use the datasets separately from DHS.

### Statistics and reproducibility

The datasets were imported to STATA® MP 4 core v17, and declaration for survey design for each dataset was done for weights, primary sampling unit and strata as per instructions by DHS in order to accommodate for stratification by province and state, size group. Dependent variables were computed and required independent variables were recoded. Association between categorical variables was assessed using design adjusted Chi square test. Further, Multinominal Logistic Regression analysis was used to derive adjusted odds ratio with Category 1 as the reference category in the dependent variable. The regression models were derived separately for men and women. Subsequently Poissons Regression analysis was used to derive adjusted prevalence ratio for similar models as it is difficult to interpret an odds ratio for a cross-sectional study as there is confusion between risk or odds leading to incorrect quantitative interpretation^26^. Moreover, the prevalence of no knowledge was higher than 10% and the odds ratio would overestimate the prevalence ratio. However, due to the limitation of Poissons regression with svy commands, in the dependent variable was converted into binomial variable combining Category 1 and Category 2 into a single category as “Knowledge about the spread of Tuberculosis” and Category 3 and Category 4 into a single category as “No knowledge about the spread of Tuberculosis”. All the statistical analysis was carried out under the subset of svy commands that has inherent property for measures similar to robust measures for poisons regression^27^. Microsoft® Excel 365 was used to make line charts. QGIS® Desktop 3.30.1 was used to make maps for prevalence ratio of Men: Women of No knowledge about spreading tuberculosis among men and women. To limit the length of the manuscript, the details on odds ratio are given in the main manuscript and details on prevalence ratio are given in the Supplementary Table No. 7 to Supplementary Table No. 10.

### Reporting summary

Further information on research design is available in the Nature Portfolio Reporting Summary linked to this article.

## Results

### Participants

In NFHS 3, 64,212 cases from Mens dataset (containing data from interview of eligible men at household) and 109,032 cases from Womens dataset (containing data from interview of eligible women at household) were included as they fulfilled inclusion criteria. Similarly, from NFHS 4, 91,293 cases from Mens dataset and 618,274 cases from Womens dataset were included for analysis. In addition to the above, from NFHS 5, 85,751 cases from Mens dataset and 691,750 cases from Womens dataset were included for analysis.

### Descriptive data

In NFHS 3, 18.26% men and 19.93% women were from the age group fifteen to nineteen years. 38.00% of the men were residing in urban area whereas 64.53% women were residing in rural areas. Most of the men and women had Secondary level of education and belonged to richest level of wealth index. More than half of them were married. While highest proportion of the men belonged from Central zone followed by South zone, most of the women were from Central zone followed by East and South zone in similar proportion. 36.41% men read newspaper or magazine, 25.23% listened to the radio, and 47.83% watched television almost every day. However, for most of the women watching television daily was the only mode of exposure to mass media on almost daily basis. More than 50% of the women never read newspaper or magazine or listened to radio. More than 75% believed that tuberculosis can be cured and would not keep a secret if family member gets tuberculosis.

In NFHS 4, the proportions for age group, level of education, wealth index, current marital status, belief that tuberculosis can be cured, belonging to zone and response for keeping secret if family member gets tuberculosis, were like those in NFHS 3 among men. However, most of the women belonged to the age group twenty to twenty-four years followed by fifteen to nineteen years. Among women similar proportions as that of NFHS 3 were seen with respect to reading newspaper or magazine, listening to radio, and watching television. However, more than 60% of both men and women resided in rural area. In addition to that, among men only 34.53% red newspaper or magazine, and only 6.98% listened to radio and 63.28% watched television almost every day. There was a major change in proportion among men for frequency of mode of exposure to mass media with respect to and listening to radio and watching television.

In NFHS 5, like that in NFHS 4, the proportion of majority of men and women remained unchanged in terms of, type of place of residence, education level, current marital status, belief that tuberculosis can be cured, and response to the question that will they keep secret if family member gets tuberculosis. Among women most of them belonged to the age group of fifteen to nineteen years. In addition to that, most of the men belonged from East zone followed by West zone. Also, there was an increase in proportion of men and women who would never read a newspaper or magazine and listen to radio. In addition to that, there were no respondents who would read newspaper or magazine, listen to radio, or watch television almost every day among both men and women who participated in NFHS 5.

The detailed distribution of eligible men and women during three NFHS surveys are given in Tables 1 and 2 respectively. Moreover, the state and union territory wise distribution for eligible men and women is given in Supplementary Table No. 1 and Supplementary Table No. 2 respectively.

**Table 1:** Univariate analysis showing distribution of eligible men during National Family Health Survey

Variable Name	Category	NFHS 3 (2005–2006) *N* = 64,212 *n* (%)	NFHS 4 (2015–2016) *N* = 91,293 *n* (%)	NFHS 5 (2019–2021) *N* = 85,751 *n* (%)
Age (in 5-year groups)	15–19 years	11,728 (18.26)	15,885 (17.40)	14,801 (17.26)
	20–24 years	11,069 (17.25)	14,954 (16.38)	13291 (15.50)
	25–29 years	10,110 (15.73)	14,379 (15.75)	13,206 (15.40)
	30–34 years	9047 (14.13)	13,046 (14.29)	12,151 (14.17)
	35–39 years	8579 (13.35)	12,306 (13.48)	11,945 (13.93)
	40–44 years	7443 (11.60)	10,745 (11.77)	9973 (11.63)
	45–49 years	6213 (9.68)	9978 (10.93)	10,384 (12.11)
Type of place of residence	Urban	24,403 (38.00)	35,595 (38.99)	30,519 (35.59)
	Rural	39,808 (62.00)	55,698 (61.01)	55,232 (64.41)
Educational Level	No education	10,283 (16.02)	9650 (10.57)	8352 (9.74)
	Primary	10,217 (15.93)	10,617 (11.63)	9647 (11.25)
	Secondary	34,985 (54.50)	53,863 (59.00)	50,061 (58.38)
	Higher	8661 (13.51)	17,163 (18.80)	17,682 (20.62)
Wealth Index	Poorest	9344 (14.57)	12,799 (14.02)	14,089 (16.43)
	Poorer	11,310 (17.62)	16,771 (18.37)	16,764 (19.55)
	Middle	12,943 (20.16)	19,190 (21.02)	18,136 (21.15)
	Richer	14,662 (22.84)	20,349 (22.29)	19,200 (22.39)
	Richest	16,004 (24.82)	22,175 (24.29)	17,570 (20.49)
Current marital status	Never in union	23,256 (36.22)	34,655 (37.96)	33,546 (39.12)
	Married	40,133 (62.50)	55,515 (60.81)	51,150 (59.65)
	Others	821 (1.28)	1123 (1.23)	1055 (1.23)
TB can be cured	Yes	55,099 (85.75)	82,757 (90.65)	78,737 (91.82)
	Don’t know	4973 (7.75)	3780 (4.14)	3396 (3.96)
	No	4137 (6.44)	4756 (5.21)	3619 (4.22)
Reads newspaper or magazine	Not at all	18,123 (28.23)	26,000 (28.48)	33,477 (39.04)
	Less than once a week	10,341 (16.11)	14,141 (15.49)	23,804 (27.76)
	At least once a week	12,295 (19.16)	19,628 (21.50)	28,469 (33.20)
	Almost every day	23,365 (36.41)	31,523 (34.53)	–
Listens to radio	Not at all	18,452 (28.72)	64,727 (70.90)	65,471 (76.35)
	Less than once a week	16,611 (25.90)	8417 (9.22)	13,797 (16.09)
	At least once a week	12,928 (20.13)	11,786 (12.91)	6483 (7.56)
	Almost every day	16,216 (25.23)	6372 (6.98)	–
Watches Television	Not at all	10,085 (15.71)	11037 (12.09)	15,015 (17.51)
	Less than once a week	12,681 (19.76)	8655 (9.48)	22,270 (25.97)
	At least once a week	10,727(16.69)	13831 (15.15)	48,466 (56.52)
	Almost every day	30,718 (47.83)	57,770 (63.28)	–
Zone	North	8764 (13.64)	13,037 (14.28)	7220 (8.42)
	Northeast	2678 (04.17)	3140 (3.44)	4708 (5.49)
	Central	16,432 (25.57)	21,399 (23.44)	10,470 (12.21)
	East	13,445 (20.98)	17,638 (19.32)	22,613 (26.37)
	West	10,476 (16.34)	16,962 (18.58)	20,503 (23.91)
	South	12,385 (19.30)	19,117 (20.94)	20,237 (23.60)
Keep secret if family member gets tuberculosis	No	52,261 (81.44)	72,231 (79.12)	65,162 (75.99)
	Yes	10,635 (16.57)	18,204 (19.94)	19,594 (22.85)
	Don’t know/Not sure	1175 (1.83)	858 (0.94)	995 (1.16)

States wise distribution for each NFHS given in Supplementary Table No. 1.

*NFH*S National Family Health Survey.

**Table 2:** Univariate analysis showing distribution of eligible women during National Family Health Survey

Variable Name	Category	NFHS 3 (2005–2006) *N* = 109,032 *n* (%)	NFHS 4 (2015–2016) *N* = 618,274 *n* (%)	NFHS 5 (2019–2021) *N* = 671,750 *n* (%)
Age (in 5-year groups)	15–19 years	21,730 (19.93)	108,507 (17.55)	113,794 (16.94)
	20–24 years	20,160 (18.49)	110,300 (17.84)	111,712 (16.63)
	25–29 years	17,968 (16.48)	101,582 (16.43)	109,092 (16.24)
	30–34 years	15,537 (14.25)	85,631 (13.85)	93,306 (13.89)
	35–39 years	13,869 (12.72)	80,005 (12.94)	90,283 (13.44)
	40–44 years	11,285 (10.35)	67,825 (10.97)	75,236 (11.20)
	45–49 years	8483 (7.78)	64,424 (10.42)	78,326 (11.66)
Type of place of residence	Urban	38,674 (35.47)	221,837 (35.88)	223,021 (33.20)
	Rural	70,358 (64.53)	396,437 (64.12)	448,729 (66.80)
Educational Level	No education	39,339 (36.08)	154,816 (25.04)	143,284 (21.33)
	Primary	15,919 (14.60)	75,491 (12.21)	77,587 (11.55)
	Secondary	44,529 (40.84)	302,212 (48.88)	341,047 (50.77)
	Higher	9235 (8.47)	85,755 (13.87)	109,831 (16.35)
Wealth Index	Poorest	16,442 (15.08)	101,521 (16.42)	120,579 (17.95)
	Poorer	19,560 (17.94)	117,719 (19.04)	132,066 (19.66)
	Middle	21,305 (19.54)	125,571 (20.31)	137,104 (20.41)
	Richer	24,260 (22.25)	133,980 (21.67)	141,739 (21.10)
	Richest	27,465 (25.19)	139,483 (22.56)	140,329 (20.89)
Current marital status	Never in union	23,137 (21.22)	144,552 (23.38)	161,287 (24.01)
	Married	81,109 (74.39)	449,114 (72.64)	482,585 (71.84)
	Others	4787 (4.39)	24,607 (3.98)	27,810 (4.14)
TB can be cured	Yes	85,732 (78.63)	548,162 (88.66)	606,053 (90.22)
	Don’t know	13,335 (12.23)	30,234 (4.89)	27,878 (4.15)
	No	9878 (9.06)	39,879 (6.45)	37,820 (5.63)
Reads newspaper or magazine	Not at all	64,765 (59.40)	353,776 (57.22)	437,511 (65.13)
	Less than once a week	16,060 (14.73)	92,061 (14.89)	134,350 (20.00)
	At least once a week	12,582 (11.54)	80,005 (12.94)	99,889 (14.87)
	Almost every day	15,526 (14.24)	92,432 (14.95)	–
Listens to radio	Not at all	57,634 (52.86)	521,143 (84.29)	582,407 (86.70)
	Less than once a week	17,870 (16.39)	33,139 (5.36)	61,264 (9.12)
	At least once a week	13,553 (12.43)	37,158 (6.01)	28,079 (4.18)
	Almost every day	19,953 (18.30)	26,833 (4.34)	–
Watches Television	Not at all	33,800 (31.00)	130,085 (21.04)	169,483 (25.23)
	Less than once a week	11,721 (10.75)	38,704 (6.26)	136,835 (20.37)
	At least once a week	12,779 (11.72)	60,900 (9.85)	365,365 (54.39)
	Almost every day	50,711 (46.51)	388,585 (62.85)	–
Zone	North	13,727 (12.59)	84,023 (13.59)	91,224 (13.58)
	Northeast	4645 (4.26)	22,567 (3.65)	24,989 (3.72)
	Central	27,585 (25.30)	160,937 (26.03)	177,073 (26.36)
	East	25,623 (23.50)	137,690 (22.27)	155,040 (23.08)
	West	16,169 (14.83)	83,282 (13.47)	89611 (13.34)
	South	21,272 (19.51)	129,714 (20.98)	133,813 (19.92)
Keep secret if family member gets tuberculosis	No	86,342 (79.19)	513,600 (83.07)	561,516 (83.59)
	Yes	18,187 (16.68)	98,058 (15.86)	104,659 (15.58)
	Don’t know/Not sure	4427 (4.06)	6677 (1.08)	5576 (0.83)

States wise distribution for each NFHS given in Supplementary Table No. 2.

*NFHS* National Family Health Survey.

Trend of Knowledge and Misconceptions about spreading to tuberculosis:

### Men

**Fig. 1:**
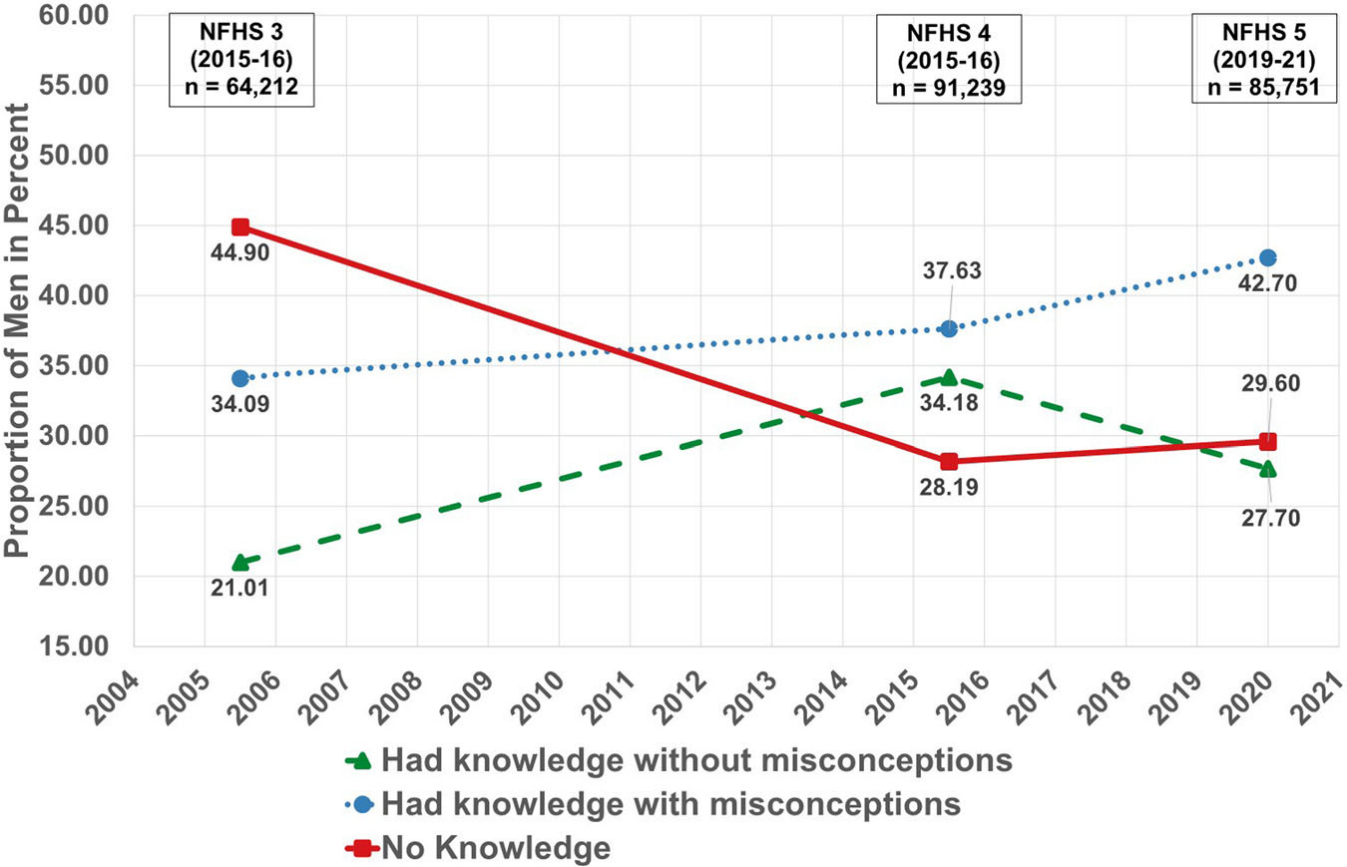
Proportion of knowledge and misconceptions about spreading of tuberculosis among men.

Across three surveys, there has been a decrease in the proportion of men who had “No knowledge” and consequently rise in proportion of those who had knowledge about spreading of tuberculosis. Moreover, during NFHS 4, the proportion of those with “No Knowledge” was less than that of those who “Had Knowledge”. In addition to that, from NFHS 4 to NFHS 5, there is an increase in proportion of those who “Had knowledge with misconceptions” but decrease in the proportion of those men who “Had knowledge without misconceptions” about the spread of tuberculosis. Hence there was an increase of misconceptions among men. (Fig. 1).

### Women

**Fig. 2:**
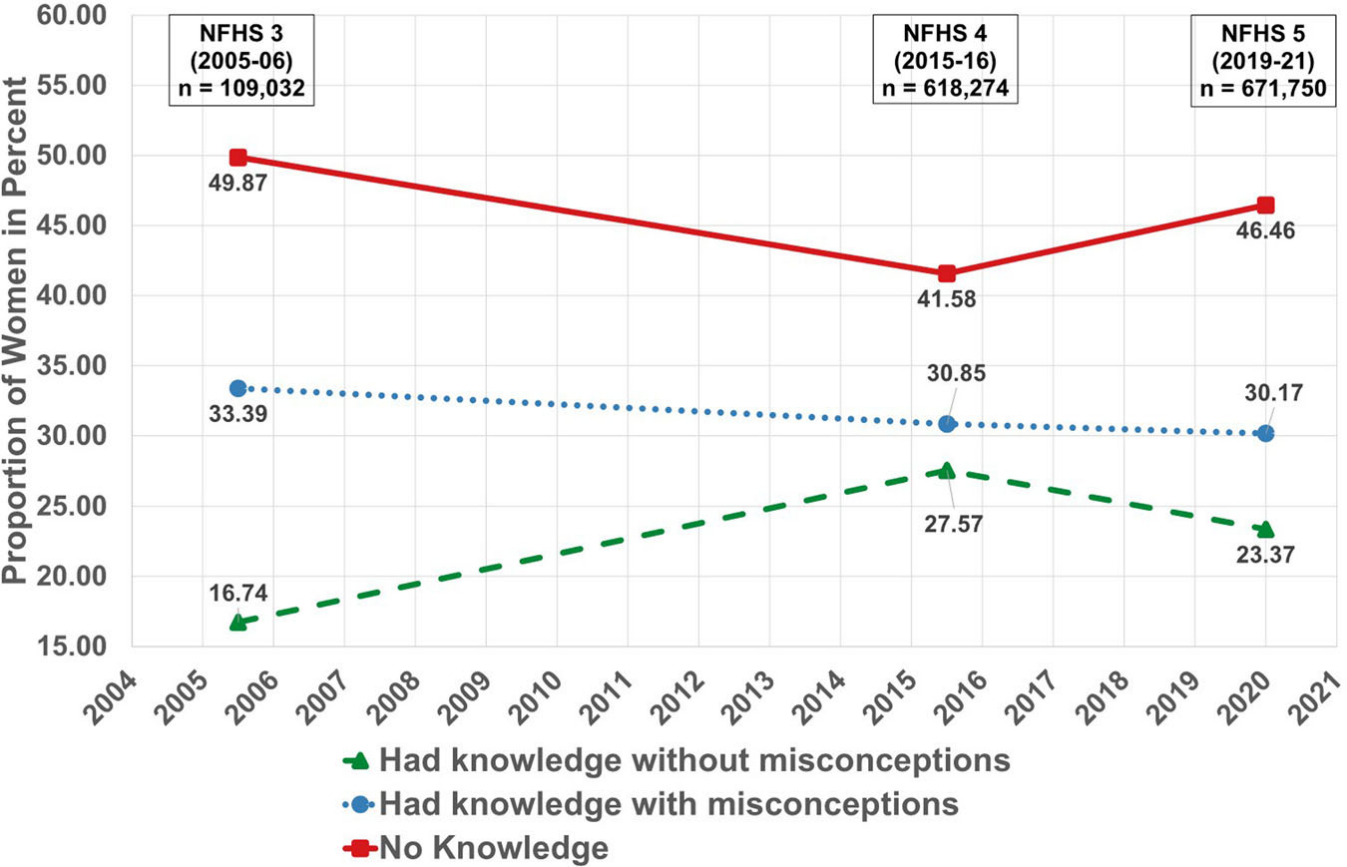
Proportion of knowledge and misconceptions about spreading of tuberculosis among women.

Across three surveys, there has been a decrease in the proportion of women who had “No knowledge” and consequently rise proportion of women who “Had knowledge” about the spread of tuberculosis. However, the proportion of women with “No Knowledge” has always been higher than that of those who “Had knowledge without misconception”. The difference between those who “Had knowledge without misconception” and those who “Had knowledge with misconceptions” about the spread of tuberculosis had been increasing. Hence, there was an increase of misconceptions among women. (Fig. 2).

**Fig. 3:**
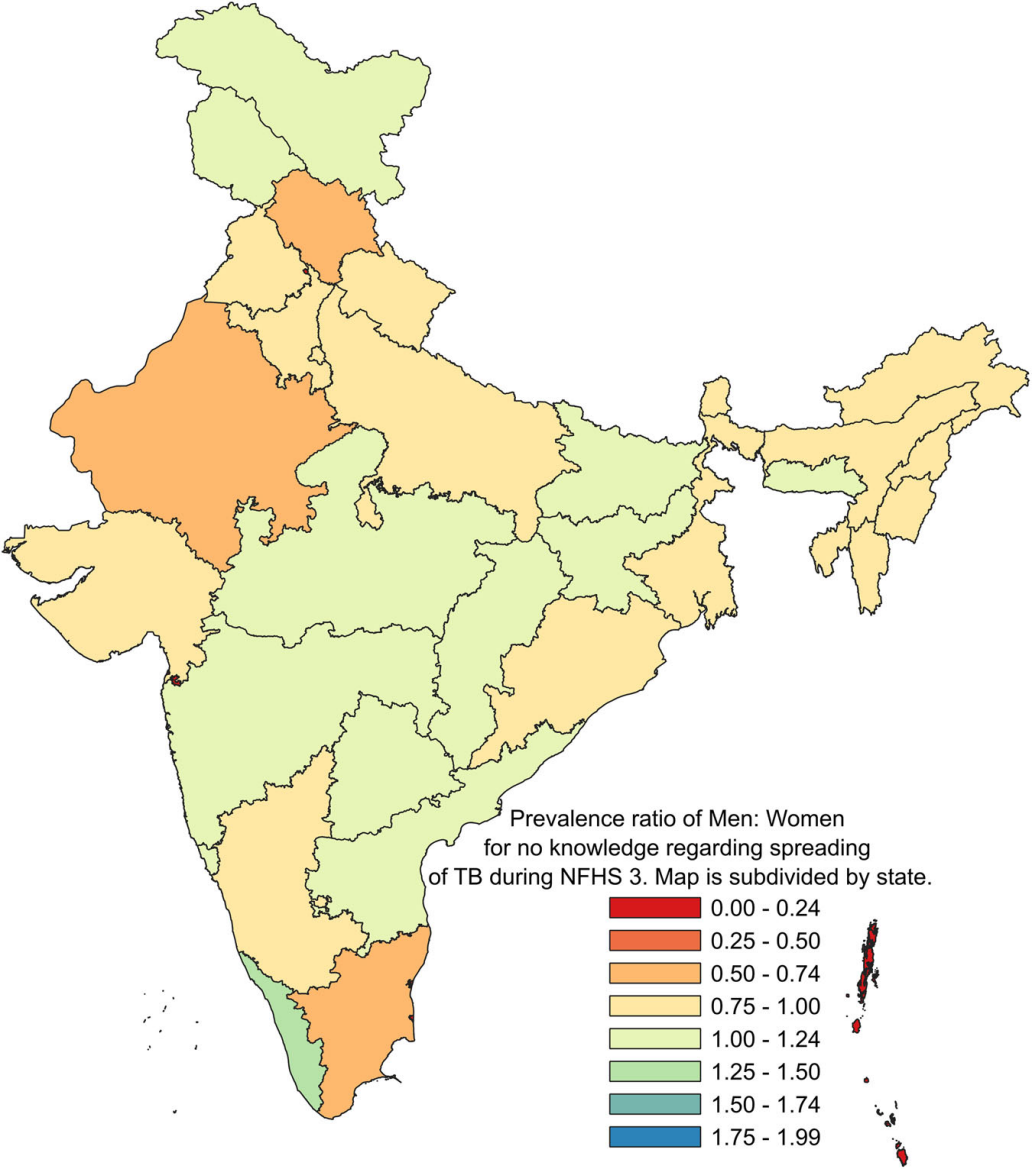
Prevalence ratio of men: women for no knowledge regarding spreading of TB during NFHS 3.

**Fig. 4:**
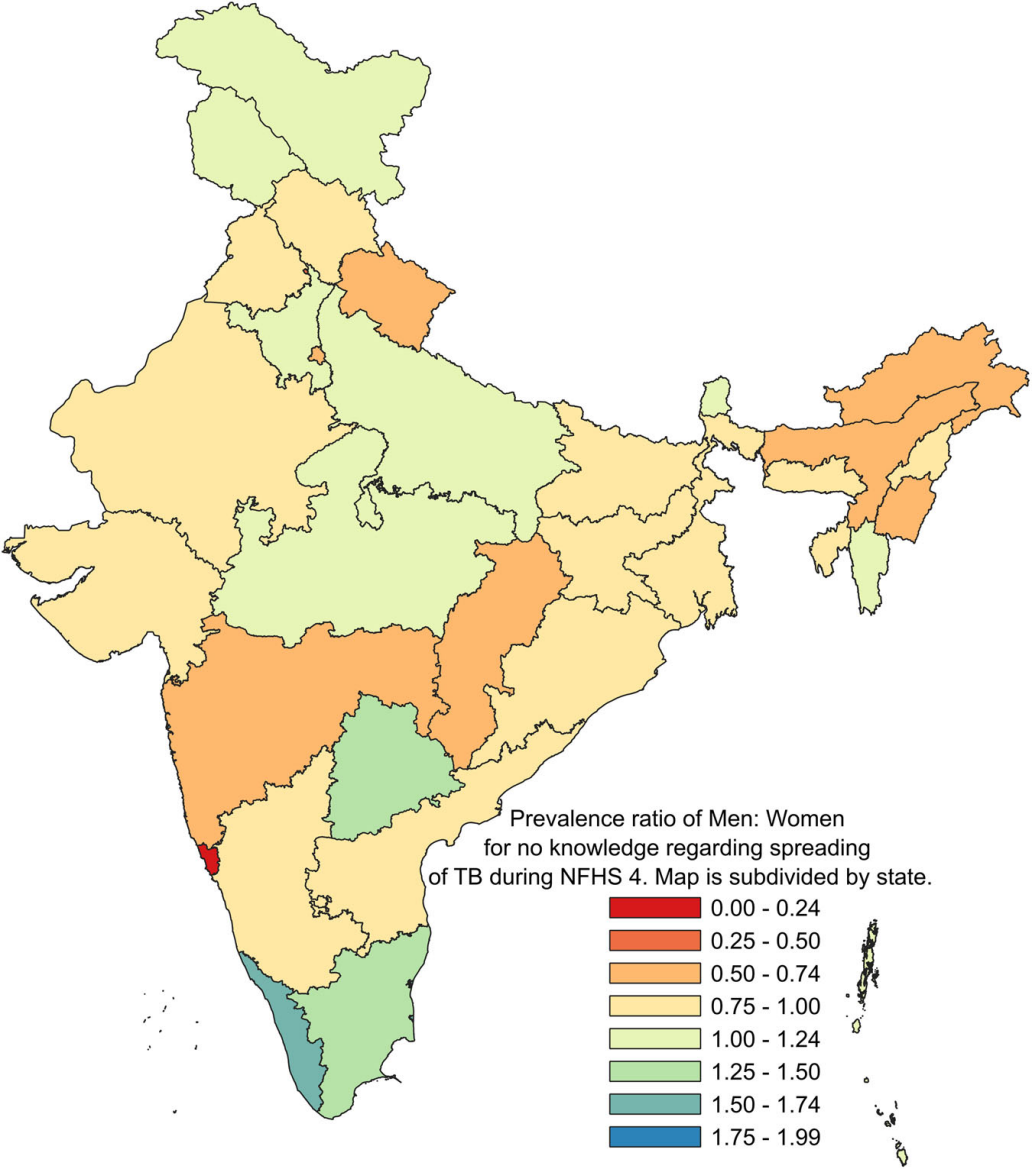
Prevalence ratio of men: women for no knowledge regarding spreading of TB during NFHS 4.

**Fig. 5:**
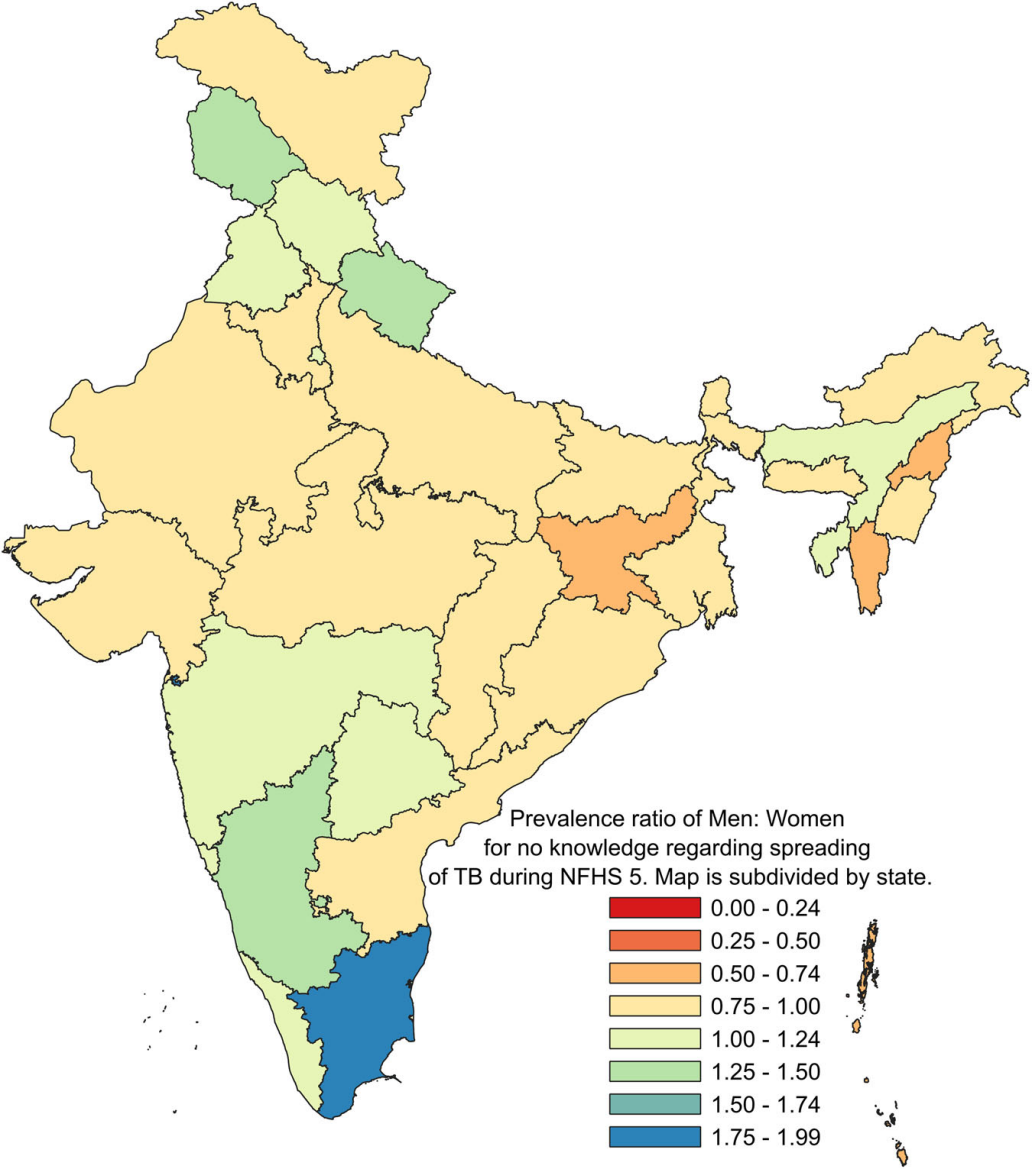
Prevalence ratio of men: women for no knowledge regarding spreading of TB during NFHS 5.

For comparison of prevalence of “No knowledge” about spreading tuberculosis among men and women across the states, during NFHS 3, NFHS 4 and NFHS 5 are presented as Prevalence Ratio on geographical basis on map of India with political boundaries denoting state and union territories. (Figs. 3, 4 and 5 respectively)

### Outcome data

Adjusted odds ratio for men and women for various factors affecting the response as “No knowledge” about spreading of tuberculosis.

### Main results

#### Age group

The Crude odds ratio for the independent variables for men and women during three rounds of NFHS is given in Supplementary Table No. 3 and Supplementary Table No. 4 respectively. While any age group was not a significant factor among men, among women the age group of fifteen to twenty four years had higher odds of having “No knowledge” about the spread of tuberculosis during NFHS 3 (aOR: 1.28 (1.12–1.44) for age group fifteen to nineteen years and 1.15 (1.03–1.28) for age group of twenty to twenty four years) and NFHS 5 (aOR: 1.07 (1.01–1.13) for both age groups) and it was statistically significant.

### Type of place of residence

Like the age groups, residing in rural areas was not a significant factor among men. However, women had the higher odds ratio of “No knowledge” who were residing in rural areas and the odds had marginal change across three surveys (aOR: 1.18 (1.07–1.30) during NFHS 3, aOR: 1.09 (1.03–1.14) during NFHS 4 and aOR: 1.12 (1.06–1.18) during NFHS 5) and it was statistically significant. It shows that there has been a disparity between men and women with respect to residing in rural areas.

### Education level

No education has constantly been associated significantly with higher odds ratio of having “No knowledge” about spread of tuberculosis among both men and women, however the odds ratio were higher among women (aOR: 2.72 (2.37–3.11) during NFHS 3, aOR: 2.08 (1.96–2.22) during NFHS 4 aOR: 1.66 (1.57–1.76) during NFHS 5) when compared to men through three surveys and the difference of odds ratio between men and women have been decreasing.

### Wealth Index

For both genders, all categories of wealth index were associated significantly with higher odds ratio of “No Knowledge” about spread of tuberculosis and the odds ratio were highest among the respondents belonging to the poorest category of wealth index during NFHS 3 and NFHS 4. However, during NFHS 5, among men only those belonging to the poorest category of wealth index were associated with higher odds ratio.

### Current marital status

For men the marital status was not a significant factor. However, among women never married had lesser odds ratio of “No knowledge” about spreading tuberculosis during NFHS 3 and NFHS 5 which was statistically significant.

### Tuberculosis can be cured

Both among men and women those who believed that tuberculosis cannot be cured, were associated with higher odds ratio of having “No knowledge” about spreading of tuberculosis. Among men the odds ratio had increased during NFHS 5 (aOR: 1.95 (1.62–2.34)) when compared to NFHS 3 (aOR: 1.74 (1.51–2.00)). However, among women the odds ratio had decreased during NFHS 5 (aOR: 1.62 (1.54–1.71)) when compared to NFHS 3 (aOR: 1.76 (1.57–1.98)) and these findings were statistically significant.

### Would keep secret if family member gets tuberculosis

For men, only during NFHS 5 had higher odds ratio (aOR: 1.21 (1.05–1.39)) for “No knowledge” about spreading of tuberculosis if they wanted keep secret if family member gets tuberculosis. However, for women, the odds ratio of “No knowledge” about spreading of tuberculosis were higher during all three surveys. Moreover, the odds ratio among women have reduced over time marginally. ((aOR 1.27 (1.16–1.38) in NFSH 3, aOR: 1.23 (1.17–1.30) in NFHS 4 and aOR: 1.26 (1.16–1.38) in NFHS 5).

### Frequency of reading newspaper or magazine

Not reading newspaper or magazine at all among men was associated with increased odds ratio of having “No knowledge” about spreading tuberculosis among men during NFHS 3 (aOR: 1.33 (1.16–1.50)) and NFHS 4 (aOR: 1.24 (1.12–1.36)) only. However, the odds ratio was insignificant during NFHS 5. Among women not reading newspaper or magazine at all was associated with increased odds ratio of having “No knowledge” about spreading of tuberculosis during three surveys. In addition to that the odds ratio had decreased from NFHS 3 (aOR: 1.23 (1.12–1.35)) to NFHS 5 (aOR: 1.16 (1.10–1.22)).

### Frequency of listening to radio

Among both genders, not listening to radio was associated with higher odds ratio of having “No knowledge” about spreading of tuberculosis during NFHS 3. How ever during NFHS 5, the odds ratio were insignificant in males and protective among females (aOR: 0.93 (0.86–0.99)).

### Frequency of watching television

For men not watching television at was associated with increased odds ratio of “No knowledge” about spread of tuberculosis during NFHS 4 (aOR: 1.14 (1.02–1.27)). However, among females, not watching television at all was associated with increased odds ratio during NFHS 3 (aOR: 1.12 (1.01–1.24)) and NFHS 5 (aOR: 1.08 (1.04–1.12)).

The detailed adjusted odds ratio for men and women are given in Tables 3 and 4 respectively.

**Table 3:** Multivariate analysis showing adjusted Odds Ratio (aOR) among men for not know spreading of tuberculosis

Variable Name	Category	NFHS 3 (2005–2006) aOR	NFHS 4 (2015–2016) aOR	NFHS 5 (2019–2021) aOR
Age Group	45–49 years	Reference Category		
	40–44 years	0.99 (0.87–1.13)	1.05 (0.93–1.17)	0.98 (0.84–1.15)
	35–39 years	0.99 (0.88–1.13)	0.98 (0.87–1.09)	0.96 (0.82–1.10)
	30–34 years	0.98 (0.87–1.12)	0.98 (0.87–1.09)	1.03 (0.88–1.20)
	25–29 years	1.10 (0.97–1.25)	0.99 (0.88–1.10)	1.00 (0.85–1.18)
	20–24 years	1.07 (0.93–1.24)	0.98 (0.86–1.10)	1.02 (0.85–1.21)
	15–19 years	1.16 (0.99–1.34)	0.99 (0.85–1.13)	0.94 (0.77–1.14)
Type of place of residence	Urban	Reference Category		
	Rural	1.08 (0.96–1.21)	1.10 (9.77–1.24)	1.16 (0.98–1.38)
Educational Level	Higher	Reference Category		
	Secondary	1.62 (1.46–1.81)***	1.32 (1.20–1.45)***	1.15 (0.99–1.33)
	Primary	1.98 (1.70–2.30)***	1.60 (1.39–1.83)***	1.43 (1.18–1.72)**
	No education	1.98 (1.65–2.37)***	1.57 (1.36–1.80)***	1.25 (1.02–1.52)**
Wealth Index	Richest	Reference Category		
	Richer	1.14 (1.02–1.26)**	1.13 (1.00–1.26)**	0.96 (0.80–1.15)
	Middle	1.30 (1.15–1.48)***	1.16 (1.01–1.31)**	1.07 (0.89–1.29)
	Poorer	1.52 (1.31–1.77)***	1.23 (1.06–1.41)***	1.00 (0.81–1.23)
	Poorest	1.70 (1.42–2.03)***	1.36 (1.17–1.58)***	1.39 (1.12–1.71)***
Current marital status	Others	Reference Category		
	Never in union	1.35 (0.98–1.86)	1.12 (0.82–1.51)	0.85 (0.61–1.18)
	Married	1.47 (1.08–2.00)	1.09 (0.76–1.35)	0.93 (0.68–1.25)
State*	Kerala	Reference Category		
Tuberculosis can be cured	Yes	Reference Category		
	No	1.74 (1.51–2.00)***	2.07 (1.81–2.35)***	1.95 (1.62–2.34)***
Keep secret if family member gets tuberculosis	Yes	1.41 (0.92–1.13)	0.99 (0.89–1.10)	1.21 (1.05–1.39)***
	No	Reference Category		
Frequency of reading newspaper or magazine	At least once a week	Reference Category		
	Less than once a week	1.13 (1.01–1.27)**	1.24 (1.03–1.26)***	1.00 (0.89–1.12)
	Not at all	1.33 (1.16–1.50)***	1.14 (1.12–1.36)**	1.00 (0.88–1.12)
Frequency of listening to radio	At least once a week	Reference Category		
	Less than once a week	1.15 (1.03–1.27)**	1.08 (0.92–1.25)	1.01 (0.70–1.02)
	Not at all	1.19 (1.06–1.32)***	1.00 (0.89–1.13)	0.85 (0.84–1.22)
Frequency of watching television	At least once a week	Reference Category		
	Less than once a week	1.00 (0.89–1.12)	1.22 (1.07–1.37)**	0.97 (0.86–1.08)
	Not at all	0.95 (0.83–1.09)	1.14 (1.02–1.27)**	1.01 (0.89–1.14)

*NFHS* National Family Health Survey, *aOR* adjusted odds ratio.

*aOR for men residing in states and union territories is given in Supplementary Table No. 3.

**p < 0.05; ***p < 0.01

**Table 4:** Multivariate analysis showing adjusted Odds Ratio (aOR) among women for having no knowledge about spreading of tuberculosis

Variable Name	Category	NFHS 3 (2005–2006) aOR	NFHS 4 (2015–2016) aOR	NFHS 5 (2019–2021) aOR
Age Group	45–49 years	Reference Category		
	40–44 years	1.01 (0.90–1.13)	0.98 (0.98–1.03)	1.00 (0.95–1.03)
	35–39 years	1.00 (0.90–1.12)	0.96 (0.91–1.00)	1.00 (0.95–1.03)
	30–34 years	1.01 (0.91–1.12)	0.95 (0.91–1.00)	0.97 (0.92–1.00)
	25–29 years	1.05 (0.94–1.17)	0.95 (0.91–1.00)	1.03 (0.98–1.06)
	20–24 years	1.15 (1.03–1.28)**	0.99 (0.94–1.05)	1.07 (1.02–1.11)**
	15–19 years	1.28 (1.12–1.44)***	1.03 (0.96–1.09)	1.07 (1.01–1.13)**
Type of place of residence	Urban	Reference Category		
	Rural	1.18 (1.07–1.30)**	1.09 (1.03–1.14)**	1.12 (1.06–1.18)***
Educational Level	Higher	Reference Category		
	Secondary	1.79 (1.61–1.97)***	1.44 (1.37–1.51)***	1.36 (1.30–1.42)***
	Primary	2.59 (2.28–2.94)***	1.95 (1.82–2.07)***	1.70 (1.61–1.79)***
	No education	2.72 (2.37–3.11)***	2.08 (1.06–2.22)***	1.67 (1.57–1.76)***
Wealth Index	Richest	Reference Category		
	Richer	1.10 (1.01–1.20)**	1.11 (1.05–1.16)***	1.07 (1.02–1.13)***
	Middle	1.28 (1.15–1.43)***	1.27 (1.19–1.34)***	1.13 (1.07–1.19)***
	Poorer	1.35 (1.19–1.53)***	1.39 (1.30–1.47)***	1.19 (1.12–1.26)***
	Poorest	1.37 (1.15–1.54)***	1.60 (1.49–1.70)***	1.31 (1.22–1.39)***
Current marital status	Others	Reference Category		
	Never in union	0.85 (0.73–0.98)**	0.98 (0.91–1.05)	0.92 (0.86–0.98)***
	Married	0.96 (0.85–1.08)	1.05 (0.99–1.11)	0.98 (0.93–1.03)
State*	Kerala	Reference Category		
Tuberculosis can be cured	Yes	Reference Category		
	No	1.76 (1.57–1.98)***	1.93 (1.83–2.03)***	1.62 (1.54–1.71)***
Keep secret if family member gets tuberculosis	Yes	1.27 (1.16–1.38)***	1.24 (1.17–1.30)***	1.22 (1.16–1.27)***
	No	Reference Category		
Frequency of reading newspaper or magazine	At least once a week	Reference Category		
	Less than once a week	1.13 (1.03–1.24)**	1.11 (1.05–1.17)***	1.07 (1.02–1.13)***
	Not at all	1.24 (1.12–1.35)***	1.15 (1.10–1.21)***	1.16 (1.10–1.22)***
Frequency of listening to radio	At least once a week	Reference Category		
	Less than once a week	1.06 (0.95–1.17)	1.04 (0.97–1.13)	1.06 (0.98–1.15)
	Not at all	1.14 (1.04–1.26)**	1.02 (0.96–1.08)	0.94 (0.86–0.99)**
Frequency of watching television	At least once a week	Reference Category		
	Less than once a week	1.13 (1.00–1.26)**	1.11 (1.05–1.17)***	1.08 (1.04–1.12)***
	Not at all	1.12 (1.01–1.24)**	1.008 (0.96–1.05)	1.09 (1.04–1.12)***

*NFHS* National Family Health Survey, *aOR* adjusted odds ratio.

*aOR for women residing in states and union territories is given in Supplementary Table No. 4. **p < 0.05 ***p < 0.01.

**p < 0.05; ***p < 0.01.

### Other analyses

In our regression model for all three surveys, we included the State or Union Territory of residence of respondents to derive aOR for residents of other states and union territories when compared to the residents of the state Kerala. During NFHS 3 for men, the highest odds ratio for “No knowledge” about spreading of tuberculosis was among those who were residing in Jharkhand (aOR: 11.02 (6.71–18.10)) followed by Madhya Pradesh (aOR: 4.96 (3.43–7.17)). Similarly, during NFHS 4 the highest odds ratio was among those who were residing in Uttarakhand (aOR: 7.33 (5.07–10.58)) followed by Himachal Pradesh (aOR: 6.04 (4.44–8.21)). However, during NFHS 5, Dadra & Nagar Haveli and Daman & Diu (aOR: 47.76 (24.25–94.07)) had the highest odds ratio followed by Bihar (aOR: 14.49 (10.02–20.97)). For women, during NFHS 3, the highest odds ratio for having “No knowledge” about spreading of tuberculosis was among those who were residing in Bihar (aOR: 15.00 (10.52–21.38)) followed by Assam (aOR: 10.07 (7.82–12.96)). Similarly, during NFHS 4, the highest odds ratio was among those who were residing in Jharkhand (aOR: 14.93 (13.18–16.90)) followed by Assam (aOR 8.49 (7.47–9.63)). However, during NFHS 5, the highest odds ratio was in those women who were residing in Bihar (aOR: 31.36 (27.65–35.57)) followed by Jharkhand (aOR: 25.46 (22.16–29.25)).

The detailed adjusted odds ratio for men and women for state and union territories is given in Supplementary Table 5 and Supplementary Table 6 respectively.

The details on unadjusted and adjusted Prevalence Ratio for Men and Women are given in Supplementary Table 7 to Supplementary Table 10.

## Discussion

India being a signatory to the 2030 Agenda for Sustainable Development^28^, we are currently implementing National Strategic Plan (NSP – 2017–2025)^29,30^ and envision tuberculosis free India by 2025. In order to achieve this goal, there is a need to adopt a comprehensive approach to gender specific and gender sensitive interventions^15^. This study was undertaken to find out gender disparity and its associated factors regarding awareness of tuberculosis in India by comparing data of three nationwide surveys viz. NFHS-3, NFHS-4 and NFHS-5.

Across the three surveys comparison, we found that there has been a decrease in the proportion of men with “no knowledge” about the spread of tuberculosis and consequently rise in proportion of those who had knowledge over the stretch of years in India. This depicts success of various strategies involved to increase public awareness viz. availability of health information sources in vernacular language and according to local needs; regular training of concerned human resources and promotion of e- learning modes. Moreover, the decrease in the proportion of women who had “no knowledge” was more as compared to men, may be due to improved access of women to electronic media via mobile and internet usage, which could not be assessed due to limitations of the study and may also be due to inclusion of females in health manpower. Moreover, the proportion of men and women with knowledge about the spread of tuberculosis was associated with misconception regarding awareness of tuberculosis transmission which can be attributed to easier access to electronic media via mobile and internet and also lack of awareness of trusted sources of correct information regarding health-related states, particularly TB. In addition, it points towards gender being an important social construct which influences the level of awareness of people about health and illness. As per social norms men have a greater public involvement and hence greater exposure to information, which leads to greater awareness about tuberculosis among men^31^. A previous study from Gujarat showed similar findings of higher proportion of men with better knowledge. It was seen that men were more aware about the mode of transmission and symptoms of tuberculosis^32^. Another study from Nanded, Maharashtra showed similar finding of higher knowledge (33.6%) and more positive attitude (53%) in men about tuberculosis compared to women^33^.

In present study, age was an important risk factor which is associated with gender disparity in awareness regarding tuberculosis transmission. Women in age group of fifteen to nineteen years age and twenty to twenty-four years of age were having “no knowledge” about the spread of tuberculosis when compared to men in same age group. Our analysis was concordant to previous similar studies which showed that women with higher age group are more aware and knowledgeable about TB^34–36^. Increase in age may add more health experience, hence, better aware about TB and identify the mode of infection. In addition to that, culturally higher aged women in India enjoy greater autonomy and freedom than younger one, thus find little or no hindrance in order to seek medical help for self thus more aware and knowledgeable than younger one^37^.

Overall, the analysis shows that the odds among those with “no education” having lesser awareness of tuberculosis transmission have reduced in both men and women but still the odds were more in women as compared to men over the years. As women with higher education have higher odds for awareness and correct knowledge regarding spread of TB, it was in the line with other studies^38,39^. It could be that educated people have greater access to various sources of information leading to more awareness about health, availability of healthcare services and use this awareness and information in accessing the health care services^40,41^.

Furthermore, women residing in rural area and belonging to low income households were acting as a risk factor for decreased awareness on transmission of tuberculosis, while, such was not the case among men, which is in line with similar other studies^42–45^. Women with better socio-economic status and those in urban areas are more likely to have better access to health, better media access to TB information, as well as good communication, transportation, and other necessities^35,46^. In addition, the rural-urban divide in knowledge and awareness can also attribute to awareness disparity depending upon the place of residence. Therefore, urbanized women and from higher socioeconomic backgrounds have a much better chance than women from rural areas and lower socioeconomic backgrounds of meeting their needs and demands thus knowledge and awareness regarding TB^47^.

It is also found that women who would like to keep it a secret if any member suffers from tuberculosis have higher odds of having no knowledge about spread of tuberculosis than men during NFHS 3 and NFHS 4 which was not so during NFHS 5. Its probable explanation could be that women with more hindrance feel lesser autonomy in terms of medical seeking behaviour thus do not easily disclose a family member’s tuberculosis^47^. During NFHS 5 there was an overlap in the odds for men and women thus eliminating the gender disparity. Usually, NFHS is completed within a year. However, NFHS 5 was completed in three years that is from 2019 to 2021. The duration of completion of survey was increased due to lockdown. However, we are of the opinion that the pandemic has not affected awareness about the methods of spreading tuberculosis in a notable way. The results can be generalised to whole population of the country as the NFHS was conducted among the nationally representative population in the country.

Various strategies to improve access to knowledge regarding tuberculosis and its transmission include creating a culture of evidence-based decision-making by the use of ICT based applications from grass root level upwards, supporting integration and improvement in TB information systems, including NIKSHAY for achievement of TB elimination goals and establishing a TB Knowledge Network (TBKN), inter-connecting all knowledge and research institutions in the country through a virtual network. The overarching role will be to establish a backbone connectivity which will enable knowledge and information sharing amongst TBKN connected institutes, enabling collaborative research, development and innovation amongst TBKN connected institutes, facilitating advanced distance education in specialized sub-areas of TB, facilitating connection between different sectoral networks in the field of research.

The key strategy is to move towards an e-learning mode utilizing the web based and mobile based learning experiences and translating the content to vernacular language and adding relevant content as per local needs at the State level. There has been also high visibility media campaign involving Amitabh Bacchan, India’s biggest film star and an ex-TB patient, as the TB brand ambassador, a big impact on conveying the threat of TB to the public at large. Moreover, TB Champions from amongst patients, technical experts, political representatives, public figures, sportsperson, and celebrities added their voice to increase visibility and action on TB. Substantial efforts have been made towards capacity building of programme managers, state IEC officers and communication facilitators with dedicated national, regional and state level trainings and workshops, to increase awareness about TB^29^.

This study has few limitations such as since it is secondary data analysis, all aspects about knowledge and awareness of tuberculosis could not be explored. Secondly, NFHS which produced the data for this study, was based on respondents’ self-reported information, with no objective validation of the information provided. Furthermore, the dataset of women used in the analysis is limited to the reproductive age group women (fifteen years to forty-nine years of age), which is insufficient to generalize the result for all the women. Similarly, elderly men dataset is not available for analysis. The proportion of men included in the survey was not comparable to that of women. Lastly, since the data for this study came from a cross-sectional survey, we were only able to look at the association between independent and dependent variables and hence any conclusions about causality could not be drawn. In the models derived using Poisson’s Regression providing Prevalence Ratio, multiple independent variables can be seen having a varied level of significance, when compared with Odds Ratio Logistic Regression providing Relative Risk Ratio. This can be attributed to the fact that Poisson’s Regression was derived after converting the dependent variable into a binomial variable for the feasibility of the statistical analysis based on the available expertise of the authors.

Based upon our study, we recommend increased usage of mass media and social media platforms for disseminating health education, since television and radio as media of communication does not hold much value in today’s era. Future research should investigate the reasons that could explain the unexplained differences in tuberculosis awareness, knowledge, and attitude amongst men and women. Moreover, frequent community health contact activities considering gender- specific needs in tuberculosis prevention and care initiatives should be promoted. Furthermore, the fear of stigma and discrimination in different ways at their homes, workplaces, healthcare settings and in communities may prevent people, women, and transgenders, from seeking healthcare. This can be tackled by adopting social behaviour change communication (SBCC) campaigns, especially targeting women, may yield greater results in tuberculosis awareness and knowledge, leading to better tuberculosis notification rates, hence, achieve the goal of ending tuberculosis in India by 2025.

### Supplementary information

Supplement File

Reporting Summary

## Data Availability

The datasets that support the findings of this study are available from DHS at https://dhsprogram.com/ but restrictions apply to the availability of these data, which were used under license for the current study. Though the datasets are available in the public domain, a formal request is required to be placed with DHS which should mention the project details such as Title, Objectives and description of tentative analysis that will be carried out. The numerical data for the Figs. 1 and 2 can be found in file named Supplementary Table 11. Further, the numerical data for Figs. 3, 4 and 5 is provided in Supplementary Tables 12, 13 and14 respectively.
