# Supplementary material for: Nationwide surveys of awareness of tuberculosis in India uncover a gender gap in tuberculosis awareness
Source: Commun Med (Lond). 2024 Aug 23;4:168. doi: 10.1038/s43856-024-00592-x (PMC11343850; doi:10.1038/s43856-024-00592-x)
Supplement: Supplementary file 1 — Supplement File [file 43856_2024_592_MOESM1_ESM.docx]

**Supplement File**

**Supplementary Figure No 1: Selection criteria for Men in current study**

**Supplementary Figure No 2: Selection criteria for Women in current study**

Supplementary Table No 1: State wise distribution of eligible men who were interviewed during National Family Health Survey.

| States and Union Territories | NFHS 3 (2005 – 2006)  N= 64,212 n (%) | NFHS 4 (2015 – 2016)  N= 91,293 n (%) | NFHS 5 (2019 – 2021)  N= 85,751 n (%) |
| --- | --- | --- | --- |
| Andaman & Nicobar Islands | – | 30.10 (0.03) | 36.00 (0.04) |
| Andhra Pradesh | 4077.50 (6.35) | 3414.40 (3.74) | 4562.00 (5.32) |
| Arunachal Pradesh | 77.10 (0.12) | 70.30 (0.08) | 30.90 (0.04) |
| Assam | 1868.60 (2.91) | 2191.00 (2.40) | 3447.20 (4.02) |
| Bihar | 4000.40 (6.23) | 5596.30 (6.13) | 9844.20 (11.48) |
| Chandigarh | – | 91.30 (0.10) | 42.00 (0.05) |
| Chhattisgarh | 1335.60 (2.08) | 2236.70 (2.45) | 1020.40 (1.19) |
| Dadra & Nagar haveli and Daman & Diu | – | 49.30 (0.054) | 65.20 (0.08) |
| Goa | 96.30 (0.15) | 356.00 (0.39) | 188.70 (0.22) |
| Gujarat | 3525.20 (5.49) | 7120.90 (7.80) | 6765.80 (7.89) |
| Haryana | 1393.40 (2.17) | 2227.50 (2.44) | 909.00 (1.06) |
| Himachal Pradesh | 359.60 (0.56) | 839.90 (0.92) | 711.70 (0.83) |
| Jammu & Kashmir | 642.10 (1.00) | 1396.80 (1.53) | 1063.30 (1.24) |
| Jharkhand | 1489.70 (2.32) | 2099.70 (2.30) | 977.60 (1.14) |
| Karnataka | 3268.40 (5.09) | 3323.10 (3.64) | 7271.70 (8.48) |
| Kerala | 1611.70 (2.51) | 2811.80 (3.08) | 3182.20 (3.71) |
| Ladakh | – | – | 18.00 (0.02) |
| Lakshadweep | – | 5.00 (0.01) | 5.90 (0.01) |
| Madhya Pradesh | 4231.60 (6.59) | 5623.60 (6.16) | 2530.50 (2.95) |
| Maharashtra | 6877.10 (10.71) | 9439.70 (10.34) | 13480.10 (15.72) |
| Manipur | 141.30 (0.22) | 136.90 (0.15) | 248.70 (0.29) |
| Meghalaya | 57.10 (0.09) | 173.50 (0.19) | 274.40 (0.32) |
| Mizoram | 147.70 (0.23) | 71.20 (0.08) | 111.50 (0.13) |
| Nagaland | 89.90 (0.14) | 87.60 (0.10) | 128.60 (0.15) |
| NCT of Delhi | 1098.00 (1.71) | 1506.30 (1.65) | 788.90 (0.92) |
| Odisha | 2221.70 (3.46) | 2757.00 (3.02) | 1337.70 (1.56) |
| Puducherry | – | 109.60 (0.12) | 38.60 (0.04) |
| Punjab | 1765.80 (2.75) | 2337.10 (2.56) | 1037.60 (1.21) |
| Rajasthan | 3499.60 (5.45) | 4646.80 (5.09) | 2650.60 (3.09) |
| Sikkim | 41.70 (0.06) | 42.00 (0.05) | 56.60 (0.07) |
| Tamilnadu | 3435.30 (5.35) | 7102.60 (7.78) | 2143.80 (2.50) |
| Telangana | – | 2300.60 (2.52) | 2992.70 (3.49) |
| Tripura | 256.80 (0.40) | 365.20 (0.40) | 411.60 (0.48) |
| Uttar Pradesh | 10318.90 (16.07) | 12817.50 (14.04) | 6590.00 (7.68) |
| Uttarakhand | 526.50 (0.82) | 730.30 (0.80) | 325.90 (0.38) |
| West Bengal | 5759.80 (8.97) | 7184.80 (7.87) | 10461.60 (12.2) |

Supplementary Table No 2: State wise distribution of eligible women who were interviewed during National Family Health Survey

| States and Union Territories | NFHS 3 (2005 – 2006) N=109,032 n (%) | NFHS 4 (2015 – 2016)  N= 618,274 n (%) | NFHS 5 (2019 – 2021)  N= 671,750 n (%) |
| --- | --- | --- | --- |
| Andaman & Nicobar Islands | – | 197.80 (0.03) | 180.00 (0.03) |
| Andhra Pradesh | 6422.00 (5.89) | 25905.70 (4.19) | 24451.70 (3.64) |
| Arunachal Pradesh | 109.00 (0.10) | 531.70 (0.08) | 497.10 (0.07) |
| Assam | 3194.60 (2.93) | 15704.20 (2.54) | 18002.90 (2.68) |
| Bihar | 9464.00 (8.68) | 46185.10 (7.47) | 57434.60 (8.55) |
| Chandigarh | – | 575.00 (0.09) | 577.70 (0.09) |
| Chhattisgarh | 2126.10 (1.95) | 15395.00 (2.49) | 16054.80 (2.39) |
| Dadra & Nagar haveli and Daman & Diu | – | 191.70 (0.03) | 248.50 (0.40) |
| Goa | 175.50 (0.16) | 865.60 (0.14) | 1074.80 (0.16) |
| Gujarat | 5386.20 (4.94) | 26585.80 (4.30) | 28683.70 (4.27) |
| Haryana | 2044.40 (1.87) | 14158.50 (2.29) | 13502.20 (2.01) |
| Himachal Pradesh | 686.90 (0.63) | 3647.80 (0.59) | 3694.60 (0.55) |
| Jammu & Kashmir | 1079.40 (0.99) | 6182.70 (1.00) | 5441.20 (0.81) |
| Jharkhand | 2551.30 (2.34) | 15889.60 (2.57) | 17331.20 (2.58) |
| Karnataka | 4939.10 (4.53) | 22415.50 (3.63) | 33587.50 (5.00) |
| Kerala | 3434.50 (3.15) | 18925.40 (3.06) | 17532.70 (2.61) |
| Ladakh | – | – | 87.30 (0.01) |
| Lakshadweep | – | 41.10 (0.01) | 41.60 (0.01) |
| Madhya Pradesh | 6378.40 (5.85) | 38456.60 (6.22) | 39767.60 (5.92) |
| Maharashtra | 10597.90 (9.72) | 55644.70 (9.00) | 59651.40 (8.88) |
| Manipur | 272.60 (0.25) | 1174.70 (0.19) | 1209.20 (0.18) |
| Meghalaya | 104.70 (0.096) | 1422.00 (0.23) | 1545.00 (0.23) |
| Mizoram | 294.40 (0.27) | 568.80 (0.09) | 550.80 (0.08) |
| Nagaland | 141.70 (0.13) | 680.10 (0.11) | 738.90 (0.11) |
| NCT of Delhi | 1494.80 (1.37) | 10139.70 (1.64) | 10748.00 (1.60) |
| Odisha | 3957.90 (3.63) | 23061.60 (3.73) | 23511.30 (3.50) |
| Puducherry | – | 741.90 (0.12) | 658.30 (0.10) |
| Punjab | 2823.90 (2.59) | 15030.20 (2.43) | 14577.00 (2.17) |
| Rajasthan | 5615.10 (5.15) | 34387.20 (5.56) | 42589.00 (6.34) |
| Sikkim | 72.00 (0.07) | 309.10 (0.05) | 315.70 (0.05) |
| Tamilnadu | 6487.40 (5.95) | 45443.10 (7.35) | 41111.10 (6.12) |
| Telangana | – | 16075.10 (2.60) | 16189.20 (2.41) |
| Tripura | 459.00 (0.42) | 2102.10 (0.34) | 2149.60 (0.32) |
| Uttar Pradesh | 18186.50 (16.68) | 101396.90 (16.40) | 115339.50 (17.17) |
| Uttarakhand | 883.20 (0.81) | 5626.30 (0.91) | 5911.40 (0.88) |
| West Bengal | 9649.30 (8.85) | 52615.10 (8.51) | 56762.90 (8.45) |

Supplementary Table No 3: Univariate analysis showing crude Odds Ratio (OR) among men residing for having no knowledge about spreading of tuberculosis.

| Variable Name | Category | NFHS 3 (2005 – 2006) OR | NFHS 4 (2015 – 2016) OR | NFHS 5 (2019 – 2021) OR |
| --- | --- | --- | --- | --- |
| Age Group | 45 – 49 years | Reference Category | | |
|  | 40 – 44 years | 0.99 (0.88 – 1.12) | 1.02 (0.92 – 1.15) | 0.99 (0.86 – 1.15) |
|  | 35 – 39 years | 0.98 (0.87 – 1.10) | 0.97 (0.86 – 1.09) | 0.97 (0.85 – 1.11) |
|  | 30 – 34 years | 0.93 (0.83 – 1.05) | 0.94 (0.84 – 1.05) | 0.99 (0.87 – 1.14) |
|  | 25 – 29 years | 0.98 (0.88 – 1.10) | 0.95 (0.86 – 1.06) | 0.99 (0.83 – 1.13) |
|  | 20 – 24 years | 0.90 (0.81 – 1.01) | 0.97 (0.87 – 1.08) | 0.95 (0.83 – 1.08) |
|  | 15 – 19 years | 1.10 (0.99 – 1.23) | 1.14 (1.03 – 1.27)*** | 1.00 (0.88 – 1.15) |
| Type of place of residence | Urban | Reference Category | | |
|  | Rural | 1.41 (1.30 – 1.52)*** | 1.67 (1.51 – 1.86)*** | 1.65 (1.42 – 1.93)*** |
| Educational Level | Higher | Reference Category | | |
|  | Secondary | 2.31 (2.09 – 2.55)*** | 1.77 (1.60 – 1.96)*** | 1.46 (1.26 – 1.69)*** |
|  | Primary | 4.28 (3.75 – 4.88)*** | 2.90 (2.53 – 3.31)*** | 2.59 (2.16 – 3.09)*** |
|  | No education | 6.72 (5.82 – 7.77)*** | 3.43 (3.01 – 3.90)*** | 2.47 (2.06 – 2.96)*** |
| Wealth Index | Richest | Reference Category | | |
|  | Richer | 1.49 (1.35 – 1.65)*** | 1.39 (1.24 – 1.56)*** | 1.10 (0.92 – 1.31) |
|  | Middle | 2.19 (1.96 – 2.44)*** | 1.73 (1.53 – 1.95)*** | 1.51 (1.26 – 1.81)*** |
|  | Poorer | 3.57 (3.15 – 4.05)*** | 2.32 (2.03 – 2.66)*** | 1.92 (1.57 – 2.35)*** |
|  | Poorest | 5.17 (4.45 – 6.01)*** | 3.42 (3.00 – 3.90)*** | 3.53 (2.90 – 4.29)*** |
| Current marital status | Others | Reference Category | | |
|  | Never in union | 0.80 (0.61 – 1.06) | 0.82 (0.62 – 1.09) | 0.68 (0.51 – 0.91)*** |
|  | Married | 0.98 (0.75 – 1.29) | 0.83 ( 0.63 – 1.10) | 0.82 (0.62 – 1.08) |
| Tuberculosis can be cured | Yes | Reference Category | | |
|  | No | 1.91 (1.67 – 2.18)*** | 1.89 (1.67 – 2.13)*** | 1.59 (1.34 – 1.89)*** |
| Keep secret if family member gets tuberculosis | Yes | 0.97 (0.87 – 1.07) | 0.89 (0.80 – 0.99)** | 0.84 (0.74 – 0.95)*** |
|  | No | Reference Category | | |
| Frequency of reading newspaper or magazine | at least once a week | Reference Category | | |
|  | less than once a week | 1.44 (1.29 – 1.61)*** | 1.29 (1.15 – 1.44)*** | 1.31 (1.17 – 1.46)*** |
|  | not at all | 2.34 (2.11 – 2.59)*** | 1.74 (1.60 – 1.89)*** | 1.85 (1.66 – 2.07)*** |
| Frequency of listening to radio | at least once a week | Reference Category | | |
|  | less than once a week | 1.34 (1.21 – 1.49)*** | 1.26 (1.08 – 1.47)*** | 1.29 (1.08 – 1.54)*** |
|  | not at all | 1.21 (1.09 – 1.35)*** | 1.21 (1.07 – 1.37)*** | 1.32 (1.11 – 1.57)*** |
| Frequency of watching television | at least once a week | Reference Category | | |
|  | less than once a week | 1.44 (1.29 – 1.61)*** | 1.43 (1.23 – 1.66)*** | 1.32 (1.18 – 1.47)*** |
|  | not at all | 1.71 (1.51 – 1.95)*** | 1.53 (1.38 – 1.70)*** | 1.95 (1.74 – 2.18)*** |
| State | Kerala | Reference Category | | |
|  | Andaman & Nicobar Islands | – | 1.01 (0.62 – 1.64) | 2.49 (1.31 – 4.75)*** |
|  | Andhra Pradesh | 1.84 (1.41 – 2.41)*** | 1.65 (1.18 – 2.31)*** | 4.82 (3.19 – 7.28)*** |
|  | Arunachal Pradesh | 8.09 (5.03 – 13.00)*** | 3.39 (2.41 – 4.77)*** | 3.13 (2.12 – 3.61)*** |
|  | Assam | 4.33 (2.83 – 6.62)*** | 5.03 (3.79 – 6.69)*** | 10.04 (7.07 – 14.26)*** |
|  | Bihar | 5.37 (3.80 – 7.60)*** | 3.89 (3.01 – 5.03)*** | 15.53 (10.87 – 22.20)*** |
|  | Chandigarh | – | 1.52 (0.35 – 6.63) | 4.13 (1.42 – 11.97)*** |
|  | Chhattisgarh | 4.55 (3.41 – 6.06)*** | 4.88 (3.69 – 6.44)*** | 2.83 (1.91 0 4.20)*** |
|  | Dadra & Nagar haveli | – | 1.94 (1.10 – 3.41)** | 47.17 (23.15 – 96.10)*** |
|  | Daman & Diu | – | 1.36 (0.64 – 2.92) |  |
|  | Goa | 1.82 (1.34 – 2.48)*** | 1.36 (0.64 – 2.92)*** | 0.58 (0.28 – 1.23) |
|  | Gujarat | 2.05 (1.54 – 2.74)*** | 0.29 (0.17 – 0.51)*** | 5.60 (3.86 – 8.13)*** |
|  | Haryana | 1.72 (1.28 – 2.31)*** | 3.33 (2.39 – 4.64)*** | 5.10 (3.41 – 7.61)*** |
|  | Himachal Pradesh | 2.02 (1.44 – 2.84)*** | 5.79 (4.28 – 7.82)*** | 8.07 (5.53 – 11.78)*** |
|  | Jammu & Kashmir | 5.70 (4.05 – 8.03)*** | 2.57 (1.96 – 3.37)*** | 2.66 (1.81 – 3.91)*** |
|  | Jharkhand | 20.74 (12.59 – 34.12)*** | 7.46 (5.61 – 9.91)*** | 11.45 (7.90 – 16.58)*** |
|  | Karnataka | 3.34 (2.55 – 4.37)*** | 3.11 (2.21 – 4.37)*** | 4.06 (2.75 – 5.98)*** |
|  | Ladakh | – | – | 2.17 (0.95 – 4.94) |
|  | Lakshadweep | – | 3.25 (1.60 – 6.57)*** | 0.78 (0.40 – 1.50) |
|  | Madhya Pradesh | 9.89 (6.83 – 14.32)*** | 3.50 (2.75 – 4.46)*** | 7.63 (5.35 – 10.8)*** |
|  | Maharashtra | 1.01 (0.79 – 1.30) | 0.71 (0.51 – 0.97)** | 2.38 (1.65 – 3.43)*** |
|  | Manipur | 0.58 (0.45 – 0.74)*** | 0.94 (0.69 – 1.27) | 0.70 (0.45 – 1.09) |
|  | Meghalaya | 5.71 (3.84 – 8.50)*** | 3.30 (2.09 – 5.21)*** | 6.38 (3.94 – 10.33)*** |
|  | Mizoram | 0.39 (0.26 – 0.58)*** | 1.26 (0.84 – 1.89) | 0.93 (0.60 – 1.43) |
|  | Nagaland | 1.28 (0.98 – 1.67)** | 2.94 (2.14 – 4.03)*** | 1.43 (0.98 – 2.10) |
|  | NCT of Delhi | 1.01 (0.71 – 1.44) | 1.46 (0.88 – 2.41) | 3.28 (2.09 – 5.16)*** |
|  | Odisha | 1.36 (0.98 – 1.89) | 2.25 (1.73 – 2.93)*** | 2.85 (2.01 – 4.05)*** |
|  | Puducherry | – | 0.96 (0.36 – 2.50) | 0.74 (0.33 – 1.65) |
|  | Punjab | 2.71 (2.03 – 3.61)*** | 3.55 (2.55 – 4.95)*** | 6.93 (4.90 – 9.81)*** |
|  | Rajasthan | 3.41 (2.47 – 4.72)*** | 4.36 (3.30 – 5.76)*** | 3.65 (2.56 – 5.19)*** |
|  | Sikkim | 1.97 (1.42 – 2.74)*** | 1.79 (1.07 – 2.98)** | 8.11 (3.12 – 21.08)*** |
|  | Tamilnadu | 0.66 (0.50 – 0.86)** | 1.87 (1.07 – 2.98)*** | 1.84 (1.30 – 2.61*** |
|  | Telangana | – | 3.19 (2.12 – 4.82)*** | 3.04 (2.11 – 4.39)*** |
|  | Tripura | 3.50 (2.26 – 5.41)*** | 5.21 (3.42 – 7.93)*** | 8.93 (5.34 – 14.93)*** |
|  | Uttar Pradesh | 4.07 (3.21 – 5.18)*** | 4.64 (3.66 – 5.88)*** | 9.21 (6.67 – 12.71)*** |
|  | Uttarakhand | 1.90 (1.36 – 2.65)*** | 6.88 (4.82 – 9.82)*** | 9.30 (5.35 – 16.16)*** |
|  | West Bengal | 7.31 (5.24 – 10.20)*** | 4.51 (3.30 – 6.16)*** | 13.02 (8.76 – 19.33)*** |

Supplementary Table No 4: Univariate analysis showing crude Odds Ratio (OR) among women residing for having no knowledge about spreading of tuberculosis.

| Variable Name | Category | NFHS 3 (2005 – 2006) OR | NFHS 4 (2015 – 2016) OR | NFHS 5 (2019 – 2021) OR |
| --- | --- | --- | --- | --- |
| Age Group | 45 – 49 years | Reference Category | | |
|  | 40 – 44 years | 1.07 (0.97 – 1.17) | 0.95 (0.91 – 0.99)** | 0.95 (0.92 – 0.99)*** |
|  | 35 – 39 years | 0.95 (0.87 – 1.04) | 0.92 (0.88 – 0.96)*** | 0.95 (0.92 – 0.99)*** |
|  | 30 – 34 years | 0.96 (0.87 – 1.06) | 0.90 (0.86 – 0.94)*** | 0.93 (0.90 – 0.97)*** |
|  | 25 – 29 years | 0.97 (0.88 – 1.07) | 0.85 (0.81 – 0.88)*** | 0.99 (0.96 – 1.03) |
|  | 20 – 24 years | 0.99 (0.89 – 1.09) | 0.83 (0.80 – 0.87)*** | 1.03 (0.99 – 1.07) |
|  | 15 – 19 years | 0.99 (0.90 – 1.10) | 0.92 (0.88 – 0.96)*** | 1.14 (1.10 – 1.18)*** |
| Type of place of residence | Urban | Reference Category | | |
|  | Rural | 2.43 (2.23 – 2.65)*** | 2.08 (1.98 – 2.18)*** | 1.95 (1.87 – 2.04)*** |
| Educational Level | Higher | Reference Category | | |
|  | Secondary | 2.94 (2.67 – 3.25)*** | 2.27 (2.17 – 2.38)*** | 1.98 (1.90 – 2.05)*** |
|  | Primary | 6.40 (5.67 – 7.22)*** | 4.18 (3.94 – 4.43)*** | 3.15 (3.01 – 3.30)*** |
|  | No education | 9.94 (8.82 – 11.20)*** | 5.63 (5.33 – 5.95)*** | 3.93 (3.76 – 4.12)*** |
| Wealth Index | Richest | Reference Category | | |
|  | Richer | 1.80 (1.65 – 1.97)*** | 1.46 (1.39 – 1.54)*** | 1.30 (1.24 – 1.36)*** |
|  | Middle | 3.08 (2.79 – 3.41)*** | 2.21 (2.10 – 2.34)*** | 1.79 (1.70 – 1.89)*** |
|  | Poorer | 4.66 (4.16 – 5.21)*** | 3.44 (3.25 – 3.65)*** | 2.74 (2.60 – 2.88)*** |
|  | Poorest | 5.85 (5.17 – 6.62)*** | 5.28 (4.98 – 5.61)*** | 4.34 (4.11 – 4.57)*** |
| Current marital status | Others | Reference Category | | |
|  | Never in union | 0.63 (0.54 – 0.72)*** | 0.79 (0.75 – 0.84)*** | 0.97 (0.92 – 1.02) |
|  | Married | 0.94 (0.84 – 1.05) | 1.01 (0.95 – 1.06) | 1.09 (1.04 – 1.15)*** |
| Tuberculosis can be cured | Yes | Reference Category | | |
|  | No | 1.97 (1.74 – 2.22)*** | 1.63 (1.55 – 1.71)*** | 2.85 (2.70 – 3.01)*** |
| Keep secret if family member gets tuberculosis | Yes | 1.11 (1.01 – 1.21)** | 0.85 (0.81 – 0.90)*** | 0.79 (0.75 – 0.82)*** |
|  | No | Reference Category | | |
| Frequency of reading newspaper or magazine | at least once a week | Reference Category | | |
|  | less than once a week | 1.41 (1.28 – 1.55)*** | 1.39 (1.32 – 1.47)*** | 1.63 (1.55 – 1.71)*** |
|  | not at all | 3.23 (2.97 – 3.52)*** | 2.44 (2.33 – 2.56)*** | 2.89 (2.76 – 3.02)*** |
| Frequency of listening to radio | at least once a week | Reference Category | | |
|  | less than once a week | 1.16 (1.05 – 1.29)** | 1.13 (1.05 – 1.22)*** | 1.48 (1.38 – 1.59)*** |
|  | not at all | 1.40 (1.28 – 1.54)*** | 1.39 (1.32 – 1.47)*** | 1.90 (1.78 – 2.03)*** |
| Frequency of watching television | at least once a week | Reference Category | | |
|  | less than once a week | 1.36 (1.21 – 1.51)*** | 1.38 (1.30 – 1.46)*** | 1.58 (1.52 – 1.64)*** |
|  | not at all | 2.05 (1.86 – 2.27)*** | 1.63 (1.56 – 1.71)*** | 2.61 (2.52 – 2.70)*** |
| State | Kerala | Reference Category | | |
|  | Andaman & Nicobar Islands | – | 1.95 (1.62 – 2.35)*** | 3.57 (2.66 – 4.79)*** |
|  | Andhra Pradesh | 3.31 (2.64 – 4.17)*** | 3.36 (2.93 – 3.85)*** | 8.75 (7.53 – 10.16)*** |
|  | Arunachal Pradesh | 10.95 (8.03 – 14.94)*** | 7.62 (6.62 – 8.77)*** | 6.37 (7.53 – 10.16)*** |
|  | Assam | 15.16 (11.42 – 20.13)*** | 14.25 (12.59 – 16.13)*** | 10.16 (9.05 – 11.41)*** |
|  | Bihar | 29.90 (20.95 – 42.65)*** | 11.06 (9.86 – 12.40)*** | 41.58 (36.69 – 47.13)*** |
|  | Chandigarh | – | 4.28 (2.90 – 6.30)*** | 4.38 (3.05 – 6.29)*** |
|  | Chhattisgarh | 6.10 (4.60 – 8.09)*** | 8.16 (7.25 – 9.18)*** | 6.17 (5.38 – 7.07)*** |
|  | Dadra & Nagar haveli | – | 4.65 (3.52 – 6.14)*** | 4.63 (3.00 – 7.13)*** |
|  | Daman & Diu | – | 4.27 – 3.31 – 5.49()*** |  |
|  | Goa | 1.59 (1.29 – 1.95)*** | 2.12 (1.63 – 2.75)*** | 0.50 (0.34 – 0.74)*** |
|  | Gujarat | 5.15 (4.11 – 6.46)*** | 5.25 (4.62 – 5.95)*** | 8.87 (7.87 – 10.00)*** |
|  | Haryana | 4.08 (3.32 – 5.01)*** | 3.96 (3.42 – 4.59)*** | 11.09 (9.72 – 12.66)*** |
|  | Himachal Pradesh | 6.74 (5.39 – 8.44)*** | 8.60 (7.57 – 9.78)*** | 7.31 (6.34 – 8.42)*** |
|  | Jammu & Kashmir | 7.16 (5.61 – 9.14)*** | 3.39 (2.99 – 3.84)*** | 2.88 (2.52 – 3.28)*** |
|  | Jharkhand | 11.97 (9.22 – 1.54)*** | 23.13 (20.46 – 26.14)*** | 31.32 (27.27 – 35.97)*** |
|  | Karnataka | 8.37 (6.71 – 10.45)*** | 8.91 (7.82 – 10.15)*** | 3.60 (3.13 – 4.13)*** |
|  | Ladakh | – | – | 2.49 (1.76 – 3.51)*** |
|  | Lakshadweep | – | 3.62 (2.93 – 4.48)*** | 1.11 (0.81 – 1.53) |
|  | Madhya Pradesh | 8.26 (6.48 – 10.52)*** | 6.52 (5.85 – 7.26)*** | 9.14 (8.17 – 10.23)*** |
|  | Maharashtra | 1.55 (1.25 – 1.93)*** | 1.47 (1.28 – 1.69)*** | 3.31 (2.95 – 3.70)*** |
|  | Manipur | 1.39 (1.12 – 1.72)*** | 2.04 (1.28 – 1.69)*** | 1.47 (1.26 – 1.72)*** |
|  | Meghalaya | 8.46 (6.24 – 11.47)*** | 5.44 (4.54 – 6.52)*** | 7.61 (6.59 – 8.79)*** |
|  | Mizoram | 0.57 (0.43 – 0.75)*** | 1.40 (1.20 – 1.63)*** | 2.05 (1.75 – 2.40)*** |
|  | Nagaland | 2.39 (1.91 – 2.99)*** | 6.07 (5.37 – 6.86)*** | 4.67 (4.08 – 5.35)*** |
|  | NCT of Delhi | 3.31 (2.58 – 4.25)*** | 4.68 (3.34 – 6.56)*** | 3.91 (3.39 – 4.50)*** |
|  | Odisha | 4.82 (3.78 – 6.13)*** | 4.15 (3.71 – 4.65)*** | 5.04 (4.48 – 5.68)*** |
|  | Puducherry | – | 1.84 (1.25 – 2.69)** | 0.54 (0.39 – 0.75)*** |
|  | Punjab | 5.26 (4.26 – 6.48)*** | 1.84 (1.25 – 2.69)*** | 8.48 (7.42 – 9.70)*** |
|  | Rajasthan | 8.09 (6.45 – 10.14)*** | 8.66 (7.72 – 9.71)*** | 7.07 (6.25 – 7.99)*** |
|  | Sikkim | 2.96 (2.35 – 3.72)*** | 1.06 (0.88 – 1.27) | 7.45 (5.92 – 9.37)*** |
|  | Tamilnadu | 2.15 (1.77 – 2.61)*** | 1.85 (1.64 – 2.10)*** | 1.27 (5.92 – 9.37)*** |
|  | Telangana | – | 3.07 (2.61 – 3.61)*** | 5.02 (4.40 – 5.73)*** |
|  | Tripura | 13.58 (10.31 – 17.88)*** | 10.70 (9.16 – 12.51)*** | 21.90 (18.66 – 25.70)*** |
|  | Uttar Pradesh | 9.14 (7.47 – 11.17)*** | 10.54 (9.49 – 11.71)*** | 20.46 (18.33 – 22.85)*** |
|  | Uttarakhand | 3.64 (2.85 – 4.66)*** | 7.82 (6.86 – 8.90)*** | 10.79 (8.94 – 13.01)*** |
|  | West Bengal | 13.40 (10.72 – 16.75)*** | 11.14 (9.84 – 13.20)*** | 25.07 (22.05 – 28.51)*** |

Supplementary Table No 5: Multivariate analysis showing adjusted Odds Ratio (aOR) among men residing in states and union territories for having no knowledge about spreading of tuberculosis.

| States and Union Territories | NFHS 3 (2005 – 2006) aOR | NFHS 4 (2015 – 2016) aOR | NFHS 5 (2019 – 2021) aOR |
| --- | --- | --- | --- |
| Kerala | Reference Category | | |
| Andaman & Nicobar Islands | – | 0.95 (0.57 – 1.57) | 2.29 (1.30 – 4.01)*** |
| Andhra Pradesh | 1.40 (1.05 – 1.85)** | 1.53 (1.08 – 2.15)** | 3.92 (2.58 – 5.93)*** |
| Arunachal Pradesh | 5.35 (3.37 – 8.47)*** | 2.31 (1.61 – 3.30)*** | 2.88 (1.94 – 4.27)*** |
| Assam | 2.93 (1.93 – 4.44)*** | 3.43 (2.57 – 4.59)*** | 8.97 (6.29 – 12.78)*** |
| Bihar | 4.07 (2.85 – 5.81)*** | 2.99 (2.27 – 3.93)*** | 14.49 (10.02 – 20.97)*** |
| Chandigarh | – | 1.92 (0.44 – 8.25) | 5.26 (1.75 – 15.82)*** |
| Chhattisgarh | 3.28 (2.44 – 4.40)*** | 4.07 (3.05 – 5.41)*** | 2.60 (1.75 – 3.85)*** |
| Dadra & Nagar haveli | – | 1.65 (0.95 – 2.87) | 47.76 (24.25 – 94.07)*** |
| Daman & Diu | – | 1.14 (0.49 – 2.64) |  |
| Goa | 1.94 (1.42 – 2.64)*** | 0.36 (0.20 – 0.62)*** | 0.61 (0.29 – 1.25) |
| Gujarat | 1.86 (1.39 – 2.49)*** | 1.81 (1.39 – 2.34)*** | 5.86 (4.06 – 8.44)*** |
| Haryana | 1.47 (1.08 – 1.99)** | 3.67 (2.63 – 5.10)*** | 5.60 (3.78 – 8.31)*** |
| Himachal Pradesh | 2.08 (1.48 – 2.92)*** | 6.04 (4.44 – 8.21)*** | 8.21 (5.69 – 11.84)*** |
| Jammu & Kashmir | 4.85 (3.41 – 6.89)*** | 2.26 (1.7 – 3.00)*** | 2.57 (1.76 – 3.75)*** |
| Jharkhand | 15.39 (9.32 – 25.40)*** | 6.04 (4.49 – 8.10)*** | 10.82 (7.43 – 15.73)*** |
| Karnataka | 2.87 (2.17 – 3.79)*** | 2.87 (2.03 – 4.04)*** | 3.65 (2.48 – 5.30)*** |
| Ladakh | – | – | 2.05 (0.89 – 4.68) |
| Lakshadweep | – | 2.12 (1.16 – 3.87)** | 0.87 (0.44 – 1.69) |
| Madhya Pradesh | 6.92 (4.76 – 10.07)*** | 2.90 (2.26 – 3.72)*** | 7.07 (4.96 – 10.05)*** |
| Maharashtra | 0.98 (0.75 – 1.25) | 0.69 (0.50 – 0.94)*** | 2.35 (1.63 – 3.39)*** |
| Manipur | 0.56 (0.43 – 0.71)*** | 0.73 (0.54 – 0.99)** | 0.64 (0.41 – 0.90)** |
| Meghalaya | 4.10 (2.63 – 6.34)*** | 2.38 (1.44 – 3.91)*** | 4.89 (3.00 – 7.98)*** |
| Mizoram | 0.41 (0.28 – 0.60)*** | 1.38 (0.91 – 2.08) | 1.03 (0.67 – 1.58) |
| Nagaland | 0.87 (0.66 – 1.13) | 2.25 (1.61 – 3.12)*** | 1.11 (0.75 – 1.63) |
| NCT of Delhi | 1.25 (0.86 – 1.80) | 1.54 (0.91 – 2.60) | 3.99 (2.52 – 6.32)*** |
| Odisha | 0.96 (0.68 – 1.34) | 1.82 (1.38 – 2.39)*** | 2.64 (1.86 – 3.74)*** |
| Puducherry | – | 1.06 (0.42 – 2.63) | 0.79 (0.34 – 1.61) |
| Punjab | 2.57 (1.92 – 3.43)*** | 3.69 (2.62 – 5.17)*** | 7.31 (5.16 – 10.34)*** |
| Rajasthan | 2.45 (1.74 – 3.44)*** | 4.05 (3.03 – 5.40)*** | 3.76 (2.66 – 5.32)*** |
| Sikkim | 1.45 (1.04 – 2.03)** | 1.61 (0.94 – 2.73) | 8.14 (3.38 – 19.61)*** |
| Tamilnadu | 0.51 (0.38 – 0.66)*** | 1.67 (1.22 – 2.28)*** | 1.57 (1.1 – 2.22)** |
| Telangana | – | 2.93 (1.91 – 4.49)*** | 2.62 (1.82 – 3.78)*** |
| Tripura | 2.50 (1.58 – 3.93)*** | 4.68 (2.97 – 7.38)*** | 8.40 (5.03 – 14.00)*** |
| Uttar Pradesh | 3.03 (2.36 – 3.88)*** | 3.95 (3.08 – 5.06)*** | 9.16 (6.67 – 12.57)*** |
| Uttarakhand | 1.72 (1.24 – 2.39)*** | 7.33 (5.07 – 10.58)*** | 10.12 (5.68 – 17.99)*** |
| West Bengal | 5.34 (3.77 – 7.55)*** | 3.80 (2.75 – 5.23)*** | 11.74 (7.97 – 17.29)*** |

Supplementary Table No 6: Multivariate analysis showing adjusted Odds Ratio (aOR) among women residing in states and union territories for having no knowledge about spreading of tuberculosis.

| States and Union Territories | NFHS 3 (2005 – 2006) aOR | NFHS 4 (2015 – 2016) aOR | NFHS 5 (2019 – 2021) aOR |
| --- | --- | --- | --- |
| Kerala | Reference Category | | |
| Andaman & Nicobar Islands | – | 1.87 (1.57 – 2.22)*** | 3.28 (2.46 – 4.38)*** |
| Andhra Pradesh | 1.99 (1.59 – 2.48)*** | 2.22 (1.93 – 2.54)*** | 6.58 (5.67 – 7.62)*** |
| Arunachal Pradesh | 6.38 (4.66 – 8.72)*** | 4.32 (3.75 – 4.97)*** | 4.77 (4.18 – 5.43)*** |
| Assam | 10.07 (7.82 – 12.96)*** | 8.49 (7.48 – 9.63)*** | 7.79 (6.94 – 8.73)*** |
| Bihar | 15.00 (10.52 – 21.38)*** | 6.22 (5.52 – 7.01)*** | 31.36 (27.65 – 35.57)*** |
| Chandigarh | – | 5.22 (3.6 – 7.55)*** | 5.35 (3.74 – 7.65)*** |
| Chhattisgarh | 3.91 (2.95 – 5.16)*** | 5.45 (4.82 – 6.15)*** | 5.13 (4.48 – 5.87)*** |
| Dadra & Nagar haveli | – | 3.43 (2.72 – 4.32)*** | 4.07 (2.51 – 6.42)*** |
| Daman & Diu | – | 3.43 (2.6 – 4.53)*** |  |
| Goa | 1.61 (1.33 – 1.93)*** | 2.26 (1.76 – 2.89)*** | 0.61 (0.43 – 0.86)*** |
| Gujarat | 3.83 (3.11 – 4.71)*** | 4.33 (3.83 – 4.90)*** | 7.83 (6.96 – 8.80)*** |
| Haryana | 3.15 (2.57 – 3.85)*** | 3.67 (3.17 – 4.25)*** | 10.99 (9.65 – 12.52)*** |
| Himachal Pradesh | 6.29 (5.09 – 7.75)*** | 8.06 (7.09 – 9.17)*** | 7.13 (6.19 – 8.19)*** |
| Jammu & Kashmir | 5.07 (4.04 – 6.34)*** | 2.52 (2.22 – 2.85)*** | 2.48 (2.18 – 2.82)*** |
| Jharkhand | 7.42 (5.75 – 9.55)*** | 14.93 (13.18 – 16.90)*** | 25.46 (22.16 – 29.25)*** |
| Karnataka | 6.36 (5.11 – 7.92)*** | 7.08 (6.14 – 8.01)*** | 3.02 (2.63 – 3.47)*** |
| Ladakh | – | – | 2.02 (1.45 – 2.81)*** |
| Lakshadweep | – | 2.80 (2.26 – 3.47)*** | 1.08 (0.79 – 1.47) |
| Madhya Pradesh | 4.84 (3.78 – 6.19)*** | 4.29 (3.78 – 4.70)*** | 7.33 (6.56 – 8.19)*** |
| Maharashtra | 1.28 (1.04 – 1.57)** | 1.25 (1.08 – 1.42)*** | 2.96 (2.65 – 3.30)*** |
| Manipur | 1.26 (1.03 – 1.52)*** | 1.46 (1.28 – 1.65)*** | 1.26 (1.08 – 1.46)*** |
| Meghalaya | 5.87 (4.43 – 7.77)*** | 3.42 (2.85 – 4.09)*** | 5.21 (4.49 – 6.05)*** |
| Mizoram | 0.64 (0.49 – 0.84)*** | 1.49 (1.28 – 1.73)*** | 2.17 (1.86 – 2.52)*** |
| Nagaland | 1.73 (1.37 – 2.17)*** | 4.09 (3.61 – 4.62)*** | 3.35 (2.92 – 3.82)*** |
| NCT of Delhi | 4.10 (3.14 – 5.32)*** | 5.04 (3.60 – 7.05)*** | 4.42 (3.83 – 5.09)*** |
| Odisha | 3.06 (2.43 – 3.85)*** | 2.74 (2.44 – 3.07)*** | 4.04 (3.59 – 4.55)*** |
| Puducherry | – | 1.60 (1.06 – 2.40)** | 0.47 (0.34 – 0.63)*** |
| Punjab | 4.78 (3.91 – 5.83)*** | 5.25 (4.49 – 6.13)*** | 8.48 (7.43 – 9.67)*** |
| Rajasthan | 4.63 (3.69 – 5.81)*** | 5.95 (5.29 – 6.67)*** | 5.93 (5.25 – 6.70)*** |
| Sikkim | 2.19 (1.71 – 2.61)*** | 0.87 (0.71 – 1.05) | 7.40 (5.9 – 9.28)*** |
| Tamilnadu | 1.39 (1.16 – 1.65)*** | 1.25 (1.10 – 1.42)*** | 0.98 (0.84 – 1.13) |
| Telangana | – | 2.13 (1.81 – 2.50)*** | 3.74 (3.27 – 4.27)*** |
| Tripura | 8.85 (6.89 – 11.37)*** | 7.89 (6.75 – 9.22)*** | 16.12 (13.78 – 18.86)*** |
| Uttar Pradesh | 5.10 (4.21 – 6.18)*** | 6.74 (6.05 – 7.50)*** | 17.02 (15.25 – 18.98)*** |
| Uttarakhand | 2.98 (2.35 – 3.77)*** | 6.86 (6.02 – 7.82)*** | 10.23 (8.49 – 12.30)*** |
| West Bengal | 8.82 (7.12 – 10.88)*** | 8.16 (7.01 – 9.49)*** | 20.23 (17.79 – 22.99)*** |

Supplementary Table No 7: Univariate analysis showing Prevalence Ratio (PR) among men residing for having no knowledge about spreading of tuberculosis.

| Variable Name | Category | NFHS 3 (2005 – 2006) PR | NFHS 4 (2015 – 2016) PR | NFHS 5 (2019 – 2021) PR |
| --- | --- | --- | --- | --- |
| Age Group | 45 – 49 years | Reference Category | | |
|  | 40 – 44 years | 1.00 (0.98 - 1.03) | 1.00 (0.97 - 1.02) | 0.98 (0.95 - 1.01) |
|  | 35 – 39 years | 1.00 (0.97 - 1.02) | 0.99 (0.97 - 1.02) | 0.98 (0.96 - 1.01) |
|  | 30 – 34 years | 1.01 (0.98 - 1.03) | 0.98 (0.96 - 1.01) | 0.97 (0.94 - 1.00) |
|  | 25 – 29 years | 1.03 (1.00 - 1.05)** | 0.97 (0.95 - 1.00) | 0.97 (0.94 - 1.00) |
|  | 20 – 24 years | 1.01 (0.99 - 1.04) | 0.98 (0.96 - 1.00) | 0.97 (0.94 - 0.99) |
|  | 15 – 19 years | 1.07 (1.40 - 1.09)*** | 1.03 (1.01 - 1.06)*** | 0.99 (0.96 - 1.02) |
| Type of place of residence | Urban | Reference Category | | |
|  | Rural | 1.15 (1.13 - 1.18)*** | 1.12 (1.10 - 1.15)*** | 1.10 (1.06 - 1.14)*** |
| Educational Level | Higher | Reference Category | | |
|  | Secondary | 1.25 (1.22 - 1.27)*** | 1.15 (1.13 - 1.17)*** | 1.11 (1.07 - 1.14)*** |
|  | Primary | 1.44 (1.40 - 1.48)*** | 1.30 (1.27 - 1.34)*** | 1.25 (1.20 - 1.30)*** |
|  | No education | 1.54 (1.40 - 1.58)*** | 1.37 (1.33 - 1.40)*** | 1.27 (1.22 - 1.32)*** |
| Wealth Index | Richest | Reference Category | | |
|  | Richer | 1.12 (1.09 - 1.14)*** | 1.09 (1.06 - 1.11)*** | 1.03 (0.99 - 1.06)*** |
|  | Middle | 1.20 (1.17 - 1.23)*** | 1.15 (1.12 - 1.18)*** | 1.10 (1.06 - 1.15)*** |
|  | Poorer | 1.30 (1.27 - 1.33)*** | 1.23 (1.19 - 1.26)*** | 1.15 (1.10 - 1.19)*** |
|  | Poorest | 1.39 (1.35 - 1.42)*** | 1.34 (1.30 - 1.37)*** | 1.29 (1.23 - 1.34)*** |
| Current marital status | Others | Reference Category | | |
|  | Never in union | 0.98 (0.93 - 1.03) | 0.95 (0.88 - 1.01) | 0.92 (0.86 - 0.98)** |
|  | Married | 0.99 (0.93 - 1.04) | 0.95 (0.89 - 1.02) | 0.95 (0.89 - 1.01) |
| Tuberculosis can be cured | Yes | Reference Category | | |
|  | No | 1.22 (1.19 - 1.25)*** | 1.24 (1.21 - 1.28)*** | 1.20 (1.16 - 1.24)*** |
| Keep secret if family member gets tuberculosis | Yes | 1.01 (0.99 - 1.03) | 1.01 (0.99 - 1.04) | 1.00 (0.97 - 1.02) |
|  | No | Reference Category | | |
| Frequency of reading newspaper or magazine | at least once a week | Reference Category | | |
|  | less than once a week | 1.08 (1.06 - 1.11)*** | 1.04 (1.02 - 1.07)*** | 1.06 (1.04 - 1.09)*** |
|  | not at all | 1.20 (1.18 - 1.22)*** | 1.17 (1.15 - 1.19)*** | 1.19 (1.16 - 1.21)*** |
| Frequency of listening to radio | at least once a week | Reference Category | | |
|  | less than once a week | 1.06 (1.04 - 1.09)*** | 1.00 (0.96 - 1.03) | 1.03 (0.99 - 1.07) |
|  | not at all | 1.08 (1.06 - 1.11)*** | 1.02 (1.00 - 1.05)** | 1.05 (1.01 - 1.09)** |
| Frequency of watching television | at least once a week | Reference Category | | |
|  | less than once a week | 1.08 (1.05 - 1.10)*** | 1.05 (1.03 - 1.08)*** | 1.04 (1.12 - 1.17)*** |
|  | not at all | 1.11 (1.09 - 1.14)*** | 1.11 (1.09 - 1.14)*** | 1.14 (1.12 - 1.17)*** |
| State | Kerala | Reference Category | | |
|  | Andaman & Nicobar Islands | – | 0.93 (0.84 - 1.03) | 1.15 (0.98 - 1.35) |
|  | Andhra Pradesh | 1.12 (1.07 - 1.18)*** | 1.04 (0.96 - 1.12) | 1.20 (1.10 - 1.31)*** |
|  | Arunachal Pradesh | 1.24 (1.17 - 1.31)*** | 1.05 (0.97 - 1.14) | 1.15 (1.06 - 1.24)*** |
|  | Assam | 1.25 (1.18 - 1.31)*** | 1.24 (1.16 - 1.32)*** | 1.38 (1.29 - 1.48)*** |
|  | Bihar | 1.26 (1.20 - 1.32)*** | 1.16 (1.09 - 1.23)*** | 1.39 (1.29 - 1.49)*** |
|  | Chandigarh | – | 0.83 (0.68 - 1.02) | 1.17 (0.89 - 1.53) |
|  | Chhattisgarh | 1.26 (1.21 - 1.32)*** | 1.06 (0.99 - 1.14) | 1.06 (0.99 - 1.14) |
|  | Dadra & Nagar haveli | – | 1.16 (1.01 - 1.32)** | 1.59 (1.45 - 1.75)*** |
|  | Daman & Diu | – | 1.05 (0.89 - 1.24) |  |
|  | Goa | 1.13 (1.06 - 1.20)*** | 0.73 (0.69 - 0.78)*** | 0.92 (0.82 - 1.03) |
|  | Gujarat | 1.12 (1.07 - 1.18)*** | 1.08 (1.02 - 1.15)*** | 1.25 (1.17 - 1.34)*** |
|  | Haryana | 1.10 (1.04 - 1.16)*** | 0.98 (0.91 - 1.05) | 1.11 (1.03 - 1.19)** |
|  | Himachal Pradesh | 1.10 (1.05 - 1.16)*** | 1.13 (1.06 - 1.22)*** | 1.28 (1.18 - 1.38)*** |
|  | Jammu & Kashmir | 1.27 (1.21 - 1.33)*** | 1.09 (1.02 - 1.16)*** | 1.11 (1.03 - 1.20)** |
|  | Jharkhand | 1.37 (1.31 - 1.44)*** | 1.21 (1.14 - 1.29)*** | 1.31 (1.21 - 1.41)*** |
|  | Karnataka | 1.17 (1.12 - 1.22)*** | 1.14 (1.05 - 1.24)** | 1.23 (1.14 - 1.33)*** |
|  | Ladakh | – | – | 1.03 (0.88 - 1.21) |
|  | Lakshadweep | – | 1.27 (1.04 - 1.57)** | 0.93 (0.83 - 1.05) |
|  | Madhya Pradesh | 1.25 (1.20 - 1.31)*** | 1.16 (1.10 - 1.23)*** | 1.25 (1.17 - 1.34)*** |
|  | Maharashtra | 1.00 (0.95 - 1.05) | 0.86 (0.81 - 0.92)*** | 1.12 (1.04 - 1.20)** |
|  | Manipur | 0.91 (0.86 - 0.95)*** | 0.88 (0.83 - 0.94)*** | 0.93 (0.87 - 1.00) |
|  | Meghalaya | 1.26 (1.19 - 1.32)*** | 1.01 (0.92 - 1.12) | 1.26 (1.14 - 1.40)*** |
|  | Mizoram | 0.83 (0.77 - 0.90)*** | 1.03 (0.94 - 1.12) | 1.01 (0.93 - 1.10) |
|  | Nagaland | 1.04 (0.99 - 1.10) | 1.23 (1.14 - 1.33)*** | 1.06 (0.98 - 1.14) |
|  | NCT of Delhi | 1.01 (1.04 - 1.16) | 0.89 (0.81 -0.98)** | 1.07 (0.98 - 1.16) |
|  | Odisha | 1.06 (0.99 - 1.14) | 1.13 (1.06 - 1.21)*** | 1.10 (1.03 - 1.18)** |
|  | Puducherry | – | 0.92 (0.77 - 1.11) | 0.92 (0.82 - 1.02) |
|  | Punjab | 1.18 (1.12 - 1.24)*** | 0.93 (0.87 - 1.00) | 1.26 (1.18 - 1.36)*** |
|  | Rajasthan | 1.14 (1.09 - 1.20)*** | 1.07 (1.00 - 1.13)** | 1.00 (0.94 - 1.07) |
|  | Sikkim | 1.08 (1.03 - 1.14)** | 0.85 (0.79 - 0.92)*** | 1.20 (0.98 - 1.47) |
|  | Tamilnadu | 0.92 (0.87 - 0.97)** | 1.03 (0.96 - 1.11) | 1.03 (0.96 - 1.10) |
|  | Telangana | – | 1.17 (1.07 - 1.30)** | 1.19 (1.10 - 1.28)*** |
|  | Tripura | 1.24 (1.16 - 1.32)*** | 1.34 (1.22 - 1.49)*** | 1.47 (1.33 - 1.62)*** |
|  | Uttar Pradesh | 1.19 (1.14 - 1.25)*** | 1.12 (1.06 - 1.19)*** | 1.24 (1.17 - 1.32)*** |
|  | Uttarakhand | 1.10 (1.04 - 1.17)*** | 1.10 (1.02 - 1.19)** | 1.27 (1.12 - 1.43)*** |
|  | West Bengal | 1.27 (1.21 - 1.35)*** | 1.17 (1.09 - 1.25)*** | 1.48 (1.37 - 1.59)*** |

Supplementary Table No 8: Univariate analysis showing Prevalence Ratio (PR) among women residing for having no knowledge about spreading of tuberculosis.

| Variable Name | Category | NFHS 3 (2005 – 2006) PR | NFHS 4 (2015 – 2016) PR | NFHS 5 (2019 – 2021) PR |
| --- | --- | --- | --- | --- |
| Age Group | 45 – 49 years | Reference Category | | |
|  | 40 – 44 years | 1.00 (0.98 - 1.02) | 0.98 (0.97 - 0.99)** | 0.98 (0.97 - 0.99)*** |
|  | 35 – 39 years | 0.99 (0.97 - 1.01) | 0.97 (0.96 - 0.97)*** | 0.97 (0.96 - 0.98)*** |
|  | 30 – 34 years | 0.99 (0.98 - 1.01) | 0.96 (0.95 - 0.97)*** | 0.97 (0.95 - 0.97)*** |
|  | 25 – 29 years | 0.99 (0.98 - 1.01) | 0.94 (0.94 - 0.95)*** | 0.96 (0.96 - 0.97)*** |
|  | 20 – 24 years | 1.00 (0.98 - 1.02) | 0.93 (0.92 - 0.94)*** | 0.96 (0.95 - 0.97)*** |
|  | 15 – 19 years | 1.02 (1.01 - 1.04)** | 0.95 (0.94 - 0.96)*** | 0.98 (0.98 - 0.99)** |
| Type of place of residence | Urban | Reference Category | | |
|  | Rural | 1.20 (1.17 - 1.22)*** | 1.18 (1.16 - 1.19)*** | 1.15 (1.14 - 1.16)*** |
| Educational Level | Higher | Reference Category | | |
|  | Secondary | 1.31 (1.28 - 1.33)*** | 1.22 (1.21 - 1.23)*** | 1.18 (1.17 - 1.19)*** |
|  | Primary | 1.54 (1.50 - 1.57)*** | 1.41 (1.40 - 1.43)*** | 1.32 (1.31 - 1.33)*** |
|  | No education | 1.63 (1.59 - 1.66)*** | 1.50 (1.49 - 1.52)*** | 1.38 (1.37 - 1.39)*** |
| Wealth Index | Richest | Reference Category | | |
|  | Richer | 1.16 (1.14 - 1.18)*** | 1.11 (1.10 - 1.12)*** | 1.07 (1.06 - 1.08)*** |
|  | Middle | 1.29 (1.26 - 1.31)*** | 1.21 (1.20 - 1.23)*** | 1.15 (1.14 - 1.16)*** |
|  | Poorer | 1.36 (1.34 - 1.39)*** | 1.33 (1.32 - 1.35)*** | 1.25 (1.24 - 1.27)*** |
|  | Poorest | 1.42 (1.39 - 1.45)*** | 1.46 (1.44 - 1.47)*** | 1.38 (1.37 - 1.40)*** |
| Current marital status | Others | Reference Category | | |
|  | Never in union | 0.91 (0.89 - 0.93)*** | 0.90 (0.89 - 0.91)*** | 0.94 (0.93 - 0.95)*** |
|  | Married | 0.97 (0.95 - 0.99)** | 0.97 (0.96 - 0.98)*** | 0.99 (0.98 - 1.00) |
| Tuberculosis can be cured | Yes | Reference Category | | |
|  | No | 1.22 (1.20 - 1.24)*** | 1.21 (1.20 - 1.22)*** | 1.13 (1.12 - 1.14)*** |
| Keep secret if family member gets tuberculosis | Yes | 1.02 (1.00 - 1.04)** | 1.00 (0.99 - 1.01) | 0.99 (0.8 - 1.00) |
|  | No | Reference Category | | |
| Frequency of reading newspaper or magazine | at least once a week | Reference Category | | |
|  | less than once a week | 1.08 (1.06 - 1.10)*** | 1.06 (1.05 - 1.07)*** | 1.07 (1.06 - 1.08)*** |
|  | not at all | 1.28 (1.26 - 1.30)*** | 1.25 (1.24 - 1.26)*** | 1.26 (1.25 - 1.27)*** |
| Frequency of listening to radio | at least once a week | Reference Category | | |
|  | less than once a week | 1.05 (1.02 - 1.07)*** | 1.00 (0.98 - 1.01) | 1.06 (1.04 - 1.07)*** |
|  | not at all | 1.12 (1.10 - 1.14)*** | 1.08 (1.07 - 1.09)*** | 1.12 (1.11 - 1.14)*** |
| Frequency of watching television | at least once a week | Reference Category | | |
|  | less than once a week | 1.05 (1.03 - 1.07)*** | 1.04 (1.03 - 1.05)*** | 1.09 (1.08 - 1.10)*** |
|  | not at all | 1.17 (1.09 - 1.13)*** | 1.10 (1.09 - 1.11)*** | 1.24 (1.23 - 1.25)*** |
| State | Kerala | Reference Category | | |
|  | Andaman & Nicobar Islands | – | 1.09 (1.04 - 1.14)*** | 1.19 (1.12 - 1.26)*** |
|  | Andhra Pradesh | 1.22 (1.18 - 1.27)*** | 1.19 (1.15 - 1.23)*** | 1.23 (1.20 - 1.14)*** |
|  | Arunachal Pradesh | 1.35 (1.30 - 1.40)*** | 1.41 (1.37 - 1.45)*** | 1.24 (1.21 - 1.27)*** |
|  | Assam | 1.41 (1.37 - 1.46)*** | 1.60 (1.56 - 1.64)*** | 1.40 (1.37 - 1.43)*** |
|  | Bihar | 1.37 (1.32 - 1.42)*** | 1.42 (1.39 - 1.45)*** | 1.57 (1.53 - 1.60)*** |
|  | Chandigarh | – | 1.11 (1.02 - 1.20)*** | 1.21 (1.11 - 1.32)*** |
|  | Chhattisgarh | 1.33 (1.28 -1.39)*** | 1.34 (1.31 - 1.37)*** | 1.21 (1.18 - 1.24)*** |
|  | Dadra & Nagar haveli | – | 1.35 (1.25 - 1.45)*** | 1.16 (1.07 - 1.26)*** |
|  | Daman & Diu | – | 1.43 (1.34 - 1.52)*** |  |
|  | Goa | 1.08 (1.04 - 1.12)*** | 1.06 (1.00 - 1.36)** | 0.90 (0.87 - 0.93)*** |
|  | Gujarat | 1.26 (1.22 - 1.30)*** | 1.35 (1.31 - 1.39)*** | 1.37 (1.34 - 1.40)*** |
|  | Haryana | 1.27 (1.23 - 1.31)*** | 1.04 (1.02 - 1.07)*** | 1.21 (1.18 - 1.23)*** |
|  | Himachal Pradesh | 1.32 (1.28 - 1.36)*** | 1.39 (1.35 - 1.42)*** | 1.29 (1.25 - 1.32)*** |
|  | Jammu & Kashmir | 1.33 (1.28 - 1.38)*** | 1.15 (1.12 - 1.18)*** | 1.08 (1.06 - 1.11)*** |
|  | Jharkhand | 1.39 (1.35 - 1.44)*** | 1.58 (1.54 - 1.62)*** | 1.63 (1.60 - 1.67)*** |
|  | Karnataka | 1.31 (1.27 - 1.36)*** | 1.39 (1.35 - 1.43)*** | 1.11 (1.08 - 1.14)*** |
|  | Ladakh | – | - | 1.07 (1.00 - 1.15)** |
|  | Lakshadweep | – | 1.35 (1.28 - 1.41)*** | 1.02 (0.97 - 1.08) |
|  | Madhya Pradesh | 1.29 (1.25 - 1.34)*** | 1.28 (1.26 - 1.31)*** | 1.30 (1.27 - 1.32)*** |
|  | Maharashtra | 1.08 (1.04 - 1.12)*** | 1.05 (1.02 - 1.08)*** | 1.17 (1.15 - 1.20)*** |
|  | Manipur | 1.06 (1.02 - 1.10)** | 1.07 (1.05 - 1.10)*** | 1.04 (1.01 - 1.06)** |
|  | Meghalaya | 1.33 (1.28 - 1.38)*** | 1.20 (1.15 - 1.25)*** | 1.32 (1.28 - 1.35)*** |
|  | Mizoram | 0.86 (0.82 - 0.91)*** | 1.08 (1.05 - 1.12)*** | 1.15 (1.12 - 1.19)*** |
|  | Nagaland | 1.16 (1.12 - 1.20)*** | 1.47 (1.43 - 1.50)*** | 1.26 (1.22 - 1.29)*** |
|  | NCT of Delhi | 1.16 (1.12 - 1.20)*** | 1.16 (1.10 - 1.22)*** | 1.09 (1.07 - 1.12)*** |
|  | Odisha | 1.31 (1.26 - 1.36)*** | 1.34 (1.31 - 1.37)*** | 1.22 (1.19 - 1.25)*** |
|  | Puducherry | – | 1.06 (0.97 - 1.15) | 0.88 (0.85 - 0.91)*** |
|  | Punjab | 1.31 (1.27 - 1.35)*** | 1.07 (1.04 - 1.10)*** | 1.30 (1.27 - 1.34)*** |
|  | Rajasthan | 1.34 (1.29 - 1.38)*** | 1.30 (1.27 - 1.32)*** | 1.15 (1.12 - 1.17)*** |
|  | Sikkim | 1.16 (1.12 - 1.20)*** | 0.95 (0.92 - 0.98)** | 1.28 (1.22 - 1.34)*** |
|  | Tamilnadu | 1.15 (1.11 - 1.19)*** | 1.04 (1.02 - 1.06)*** | 0.96 (0.94 - 0.98)*** |
|  | Telangana | – | 1.18 (1.14 - 1.22)*** | 1.17 (1.14 - 1.20)*** |
|  | Tripura | 1.44 (1.39 - 1.49)*** | 1.56 (1.51 - 1.61)*** | 1.44 (1.39 - 1.48)*** |
|  | Uttar Pradesh | 1.33 (1.29 - 1.37)*** | 1.19 (1.17 - 1.22)*** | 1.35 (1.33 - 1.38)*** |
|  | Uttarakhand | 1.24 (1.19 - 1.29)*** | 1.32 (1.29 - 1.36)*** | 1.22 (1.18 - 1.26)*** |
|  | West Bengal | 1.39 (1.35 - 1.44)*** | 1.41 (1.14 - 1.22)*** | 1.61 (1.57 - 1.64)*** |

Supplementary Table No 9: Multivariate analysis showing adjusted Prevalence Ratio (aPR) among men residing for having no knowledge about spreading of tuberculosis.

| Variable Name | Category | NFHS 3 (2005 – 2006) aPR | NFHS 4 (2015 – 2016) aPR | NFHS 5 (2019 – 2021) aPR |
| --- | --- | --- | --- | --- |
| Age Group | 45 – 49 years | Reference Category |  |  |
|  | 40 – 44 years | 1.00 (0.98 - 1.01) | 1.00 (0.98 - 1.03) | 0.98 (0.96 - 1.01) |
|  | 35 – 39 years | 1.00 (0.98 - 1.01) | 1.00 (0.98 - 1.02) | 0.99 (0.96 - 1.01) |
|  | 30 – 34 years | 1.00 (0.99 - 1.01) | 1.00 (0.98 - 1.03) | 0.99 (0.96 - 1.02) |
|  | 25 – 29 years | 1.01 (1.00 - 1.03)** | 1.00 (0.98 - 1.02) | 0.98 (0.95 - 1.01) |
|  | 20 – 24 years | 1.01 (0.99 - 1.03) | 1.01 (0.99 - 1.03) | 0.99 (0.95 - 1.02) |
|  | 15 – 19 years | 1.02 (1.01 - 1.04)** | 1.04 (1.01 - 1.06)*** | 0.98 (0.94 - 1.01) |
| Type of place of residence | Urban | Reference Category |  |  |
|  | Rural | 1.00 (0.99 - 1.02) | 1.02 (1.00 - 1.04)** | 1.02 (0.99 - 1.06) |
| Educational Level | Higher | Reference Category |  |  |
|  | Secondary | 1.06 (1.05 - 1.08)*** | 1.06 (1.05 - 1.08)*** | 1.04 (1.01 - 1.07)** |
|  | Primary | 1.09 (1.07 - 1.11)*** | 1.12 (1.09 - 1.15)*** | 1.08 (1.04 - 1.05)*** |
|  | No education | 1.09 (1.07 - 1.11)*** | 1.13 (1.10 - 1.16)*** | 1.08 (1.04 - 1.12)*** |
| Wealth Index | Richest | Reference Category |  |  |
|  | Richer | 1.01 (1.00 - 1.03)** | 1.02 (1.00 - 1.04)** | 0.99 (0.95 - 1.02) |
|  | Middle | 1.03 (1.01 - 1.05)*** | 1.03 (1.01 - 1.06)** | 1.02 (0.98 - 1.06) |
|  | Poorer | 1.05 (1.03 - 1.06)*** | 1.05 (1.02 - 1.08)*** | 1.00 (0.96 - 1.05) |
|  | Poorest | 1.05 (1.03 - 1.07)*** | 1.08 (1.05 - 1.11)*** | 1.07 (1.02 - 1.12)** |
| Current marital status | Others | Reference Category |  |  |
|  | Never in union | 1.03 (0.99 - 1.06) | 1.01 (0.94 - 1.07) | 0.98 (0.92 - 1.05) |
|  | Married | 1.04 (1.00 - 1.07)** | 1.00 0.94 - 1.07) | 0.98 (0.93 - 1.05) |
| Tuberculosis can be cured | Yes | Reference Category |  |  |
|  | No | 1.08 (1.06 - 1.10)*** | 1.21 (1.18 - 1.24)*** | 1.21 (1.16 - 1.25)*** |
| Keep secret if family member gets tuberculosis | Yes | 1.00 (0.98 - 1.01) | 1.01 (0.99 - 1.03) | 1.04 (1.01 - 1.07)** |
|  | No | Reference Category |  |  |
| Frequency of reading newspaper or magazine | at least once a week | Reference Category |  |  |
|  | less than once a week | 1.01 (1.00 - 1.03)** | 1.02 (1.00 - 1.04)** | 1.02 (0.99 - 1.04) |
|  | not at all | 1.03 (1.02 - 1.05)*** | 1.07 (1.05 - 1.09)*** | 1.06 (1.03 - 1.08)*** |
| Frequency of listening to radio | at least once a week | Reference Category |  |  |
|  | less than once a week | 1.01 (1.00 - 1.02)** | 0.97 (0.95 - 1.00) | 0.99 (0.96 - 1.03) |
|  | not at all | 1.02 (1.01 - 1.03)*** | 0.99 (0.97 - 1.01) | 0.97 (0.93 - 1.00) |
| Frequency of watching television | at least once a week | Reference Category |  |  |
|  | less than once a week | 1.00 (0.99 - 1.01) | 1.03 (1.00 - 1.05)*** | 0.99 (0.97 - 1.01) |
|  | not at all | 0.99 (0.97 - 1.00) | 1.04 (1.01 - 1.06)*** | 1.01 (0.99 - 1.04) |
| State | Kerala | Reference Category |  |  |
|  | Andaman & Nicobar Islands | – | 0.91 (0.83 - 1.00) | 1.09 (0.95 - 1.24) |
|  | Andhra Pradesh | 1.07 (1.02 - 1.12)** | 1.00 (0.93 - 1.08) | 1.12 (1.42 - 1.65)** |
|  | Arunachal Pradesh | 1.16 (1.11 - 1.22)*** | 0.94 (0.88 - 1.01) | 1.07 (1.00 - 1.16)** |
|  | Assam | 1.17 (1.11 - 1.23)*** | 1.10 (1.03 - 1.17)** | 1.29 (1.20 - 1.38)*** |
|  | Bihar | 1.21 (1.15 - 1.26)*** | 1.06 (1.00 - 1.12)** | 1.32 (1.23 - 1.42)*** |
|  | Chandigarh | – | 0.88 (0.72 - 1.07) | 1.20 (0.92 - 1.56) |
|  | Chhattisgarh | 1.19 (1.14 - 1.25)*** | 1.01 (0.95 - 1.21) | 1.02 (0.95 - 1.09) |
|  | Dadra & Nagar haveli | – | 1.08 (0.95 - 1.21) | 1.53 (1.42 - 1.65)*** |
|  | Daman & Diu | – | 0.98 (0.82 - 1.18) |  |
|  | Goa | 1.12 (1.06 - 1.19)*** | 0.77 (0.73 - 0.81)*** | 0.92 (0.83 - 1.03) |
|  | Gujarat | 1.10 (1.05 - 1.15)*** | 1.04 (0.99 - 1.10) | 1.23 (1.16 - 1.31)*** |
|  | Haryana | 1.07 (1.02 - 1.12)** | 0.99 (0.93 - 1.06) | 1.11 (1.04 - 1.19)** |
|  | Himachal Pradesh | 1.11 (1.05 - 1.16)*** | 1.14 (1.06 - 1.21)*** | 1.26 (1.18 - 1.35)*** |
|  | Jammu & Kashmir | 1.22 (1.17 - 1.28)*** | 1.04 (0.98 - 1.11) | 1.09 (1.01 - 1.17)** |
|  | Jharkhand | 1.30 (1.24 - 1.36)*** | 1.12 (1.06 - 1.19) | 1.26 (1.17 - 1.35)*** |
|  | Karnataka | 1.14 (1.10 -1.19)*** | 1.09 (1.01 - 1.18)** | 1.18 (1.10 - 1.27)*** |
|  | Ladakh | – | – | 1.01 (0.87 - 1.18) |
|  | Lakshadweep | – | 1.13 (0.97 - 1.31) | 0.94 (0.84 - 1.04) |
|  | Madhya Pradesh | 1.19 (1,14 - 1.24)*** | 1.08 (1.03 - 1.14)** | 1.19 (1.12 - 1.27)*** |
|  | Maharashtra | 1.00 (0.95 - 1.04) | 0.86 (0.81 - 0.91)*** | 1.10 (1.02 - 1.18)** |
|  | Manipur | 0.91 (0.87 - 0.95)*** | 0.84 (0.79 - 0.89)*** | 0.91 (0.85 - 0.97)** |
|  | Meghalaya | 1.18 (1.12 - 1.24)*** | 0.93 (0.85 - 1.02) | 1.14 (1.03 - 1.26)** |
|  | Mizoram | 0.84 (0.78 - 0.90)*** | 1.05 (0.96 - 1.14) | 1.02 (0.94 - 1.11) |
|  | Nagaland | 0.99 (0.95 - 1.04) | 1.12 (1.04 - 1.20)** | 0.97 (0.90 - 1.04) |
|  | NCT of Delhi | 1.04 (0.99 - 1.10) | 0.90 (0.82 - 0.98)** | 1.09 (1.01 - 1.19)** |
|  | Odisha | 1.01 (0.95 - 1.08) | 1.05 (0.99 -1.12) | 1.05 (0.99 - 1.13) |
|  | Puducherry | – | 0.94 (0.79 - 1.10) | 0.91 (0.82 - 1.01) |
|  | Punjab | 1.16 (1.11 - 1.22)*** | 0.94 (0.88 - 1.01) | 1.24 (1.16 - 1.32)*** |
|  | Rajasthan | 1.10 (1.05 - 1.15)*** | 1.03 (0.97 - 1.10) | 1.00 (0.94 - 1.06) |
|  | Sikkim | 1.04 (0.99 - 1.09) | 0.83 (0.77 - 0.90)*** | 1.17 (0.99 - 1.38) |
|  | Tamilnadu | 0.90 (0.85 - 0.94)*** | 0.99 (0.93 - 1.06) | 0.98 (0.92 - 1.05) |
|  | Telangana | – | 1.12 (1.02 - 1.23)** | 1.13 (1.05 - 1.21)** |
|  | Tripura | 1.16 (1.09 - 1.24)*** | 1.28 (1.16 - 1.42)*** | 1.39 (1.26 - 1.53)*** |
|  | Uttar Pradesh | 1.14 (1.10 - 1.19)*** | 1.06 (1.01 - 1.12)** | 1.21 (1.14 - 1.28)*** |
|  | Uttarakhand | 1.08 (1.03 - 1.14)** | 1.11 (1.03 - 1.20)** | 1.26 (1.11 - 1.42)*** |
|  | West Bengal | 1.21 (1.15 - 1.26)*** | 1.10 (1.03 - 1.18)** | 1.39 (1.29 - 1.49)*** |

Supplementary Table No 10: Multivariate analysis showing adjusted Prevalence Ratio (aPR) among women residing for having no knowledge about spreading of tuberculosis.

| Variable Name | Category | NFHS 3 (2005 – 2006) aPR | NFHS 4 (2015 – 2016) aPR | NFHS 5 (2019 – 2021) aPR |
| --- | --- | --- | --- | --- |
| Age Group | 45 – 49 years | Reference Category | | |
|  | 40 – 44 years | 1.00 (0.99 - 1.01) | 1.00 (0.99 -1.00) | 0.99 (0.98 - 1.00) |
|  | 35 – 39 years | 1.00 (0.99 - 1.01) | 0.99 (0.98 - 0.99)** | 0.99 (0.98 - 1.00) |
|  | 30 – 34 years | 1.00 (0.99 - 1.01) | 0.99 (0.99 - 1.00) | 0.98 (0.98 - 0.99)** |
|  | 25 – 29 years | 1.00 (0.99 - 1.01) | 1.00 (0.99 - 1.01) | 1.00 (0.99 - 1.01) |
|  | 20 – 24 years | 1.01 (1.00 - 1.02)*** | 1.01 (1.00 - 1.02)** | 1.01 (1.00 - 1.02)*** |
|  | 15 – 19 years | 1.03 (1.01 - 1.04)*** | 1.02 (1.01 - 1.03)*** | 1.02 (1.01 - 1.03)*** |
| Type of place of residence | Urban | Reference Category | | |
|  | Rural | 1.01 (1.00 - 1.02)** | 1.01 (1.00 - 1.02)*** | 1.02 (1.01 - 1.03)*** |
| Educational Level | Higher | Reference Category | | |
|  | Secondary | 1.07 (1.06 - 1.09*** | 1.08 (1.08 - 1.09)*** | 1.07 (1.07 - 1.08)*** |
|  | Primary | 1.12 (1.10 - 1.14)*** | 1.17 (1.16 - 1.18)*** | 1.14 (1.12 - 1.15)*** |
|  | No education | 1.13 (1.11 - 1.14)*** | 1.20 (1.19 - 1.21)*** | 1.15 (1.14 - 1.16)*** |
| Wealth Index | Richest | Reference Category | | |
|  | Richer | 1.01 (1.00 - 1.02)** | 1.02 (1.01 - 1.03)*** | 1.01 (1.00 - 1.02)** |
|  | Middle | 1.03 (1.02 - 1.04)*** | 1.05 (1.04 - 1.06)*** | 1.03 (1.02 - 1.04)*** |
|  | Poorer | 1.03 (1.02 - 1.05)*** | 1.08 (1.06 - 1.09)*** | 1.05 (1.04 - 1.06)*** |
|  | Poorest | 1.03 (1.02 - 1.05)*** | 1.11 (1.10 - 1.13)*** | 1.07 (1.06 - 1.09)*** |
| Current marital status | Others | Reference Category | | |
|  | Never in union | 0.98 (0.97 - 1.00) | 0.99 (0.98 - 1.00) | 1.00 (0.99 - 1.01) |
|  | Married | 0.99 (0.98 - 1.00) | 1.01 (1.00 - 1.02) | 0.98 (0.96 - 0.99)** |
| Tuberculosis can be cured | Yes | Reference Category | | |
|  | No | 1.08 (1.07 - 1.09)*** | 1.19 (1.18 - 1.20)*** | 1.16 (1.15 - 1.17)*** |
| Keep secret if family member gets tuberculosis | Yes | 1.02 (1.01 - 1.03)*** | 1.04 (1.03 - 1.06)*** | 1.06 (1.05 - 1.07)*** |
|  | No | Reference Category | | |
| Frequency of reading newspaper or magazine | at least once a week | Reference Category | | |
|  | less than once a week | 1.01 (1.00 - 1.02)** | 1.02 (1.01 - 1.03)*** | 1.01 (1.00 - 1.01)** |
|  | not at all | 1.03 (1.02 - 1.05)*** | 1.07 (1.06 - 1.08)*** | 1.06 (1.05 - 1.07)*** |
| Frequency of listening to radio | at least once a week | Reference Category | | |
|  | less than once a week | 1.00 (0.99 - 1.01) | 0.99 (0.97 - 1.00) | 1.00 (0.98 - 1.01) |
|  | not at all | 1.02 (1.01 - 1.03)*** | 1.00 (0.99 - 1.01) | 0.98 (0.96 - 0.99)** |
| Frequency of watching television | at least once a week | Reference Category | | |
|  | less than once a week | 1.01 (1.00 - 1.02)** | 1.01 (1.00 - 1.02)** | 1.02 (1.01 - 1.02)*** |
|  | not at all | 1.00 (1.00 - 1.01)** | 1.01 (1.00 - 1.02)** | 1.05 (1.04 - 1.06)*** |
| State | Kerala | Reference Category | | |
|  | Andaman & Nicobar Islands | – | 1.05 (1.01 - 1.09)** | 1.14 (1.08 - 1.20)*** |
|  | Andhra Pradesh | 1.13 (1.09 - 1.16)*** | 1.05 (1.02 - 1.08)*** | 1.12 (1.09 - 1.15)*** |
|  | Arunachal Pradesh | 1.23 (1.19 - 1.27)*** | 1.16 (1.14 - 1.19)*** | 1.11 (1.09 - 1.14)*** |
|  | Assam | 1.30 (1.27 - 1.34)*** | 1.32 (1.29 - 1.34)*** | 1.25 (1.23 - 1.28)*** |
|  | Bihar | 1.23 (1.20 - 1.27)*** | 1.17 (1.15 - 1.20)*** | 1.38 (1.36 - 1.41)*** |
|  | Chandigarh | – | 1.14 (1.05 - 1.22)** | 1.25 (1.15 - 1.35)*** |
|  | Chhattisgarh | 1.23 (1.90 - 1.27)*** | 1.17 (1.14 - 1.19)*** | 1.12 (1.10 - 1.15)*** |
|  | Dadra & Nagar haveli | – | 1.19 (1.13 - 1.26)*** | 1.09 (1.00 - 1.18)** |
|  | Daman & Diu | – | 1.28 (1.20 - 1.36)*** |  |
|  | Goa | 1.07 (1.03 - 1.10)*** | 1.07 (1.01 - 1.12)** | 0.94 (0.91 - 0.97)*** |
|  | Gujarat | 1.20 (1.16 - 1.23)*** | 1.23 (1.20 - 1.27)*** | 1.28 (1.26 - 1.31)*** |
|  | Haryana | 1.20 (1.16 - 1.24)*** | 1.01 (0.98 - 1.03) | 1.18 (1.15 - 1.20)*** |
|  | Himachal Pradesh | 1.28 (1.25 0 1.32)*** | 1.32 (1.29 - 1.35)*** | 1.26 (1.23 - 1.29)*** |
|  | Jammu & Kashmir | 1.24 (1.20 - 1.28)*** | 1.05 (1.02 - 1.07)*** | 1.02 (1.00 - 1.04)** |
|  | Jharkhand | 1.28 (1.24 - 1.32)*** | 1.34 (1.31 - 1.37)*** | 1.48 (1.45 - 1.51)*** |
|  | Karnataka | 1.25 (1.21 - 1.28)*** | 1.26 (1.23 - 1.30)*** | 1.04 (1.02 - 1.06)*** |
|  | Ladakh | – | – | 1.00 (0.94 - 1.06) |
|  | Lakshadweep | – | 1.21 (1.16 - 1.27)*** | 1.00 (0.95 - 1.06) |
|  | Madhya Pradesh | 1.19 (1.16 - 1.23)*** | 1.11 (1.09 - 1.13)*** | 1.19 (1.16 - 1.21)*** |
|  | Maharashtra | 1.05 (1.01 - 1.08)** | 0.99 (0.96 - 1.01) | 1.12 (1.10 - 1.14)*** |
|  | Manipur | 1.04 (1.01 - 1.08)** | 0.99 (0.96 - 1.01) | 0.98 (0.96 - 1.01) |
|  | Meghalaya | 1.24 (1.20 - 1.28)*** | 1.05 (1.01 - 1.09)** | 1.16 (1.13 - 1.20)*** |
|  | Mizoram | 0.87 (0.83 - 0.92)*** | 1.08 (1.05 - 1.11)*** | 1.16 (1.12 - 1.19)*** |
|  | Nagaland | 1.10 (1.06 - 1.14)*** | 1.26 (1.23 - 1.29)*** | 1.12 (1.09 - 1.15)*** |
|  | NCT of Delhi | 1.19 (1.15 - 1.23)*** | 1.15 (1.09 - 1.21)*** | 1.11 (1.08 - 1.14)*** |
|  | Odisha | 1.20 (1.16 - 1.24)*** | 1.16 (1.13 - 1.18)*** | 1.12 (1.09 - 1.14)*** |
|  | Puducherry | – | 1.01 (0.93 - 1.10) | 0.85 (0.83 - 0.88)*** |
|  | Punjab | 1.26 (1.22 - 1.30)*** | 1.05 (1.03 - 1.08)*** | 1.27 (1.24 - 1.30)*** |
|  | Rajasthan | 1.22 (1.18 - 1.25)*** | 1.14 (1.12 - 1.16)*** | 1.07 (1.05 - 1.09)*** |
|  | Sikkim | 1.10 (1.07 - 1.14)*** | 0.89 (0.87 - 0.92)*** | 1.25 (1.20 - 1.30)*** |
|  | Tamilnadu | 1.07 (1.04 - 1.11)*** | 0.94 (0.92 - 0.96)*** | 0.89 (0.87 - 0.91)*** |
|  | Telangana | – | 1.06 (1.02 - 1.09)*** | 1.06 (1.04 - 1.09)*** |
|  | Tripura | 1.32 (1.28 - 1.36)*** | 1.37 (1.33 - 1.41)*** | 1.28 (1.24 - 1.31)*** |
|  | Uttar Pradesh | 1.21 (1.18 - 1.24)*** | 1.04 (1.02 - 1.06)*** | 1.24 (1.22 - 1.26)*** |
|  | Uttarakhand | 1.18 (1.14 - 1.22)*** | 1.24 (1.21 - 1.27)*** | 1.17 (1.14 - 1.21)*** |
|  | West Bengal | 1.29 (1.25 - 1.32)*** | 1.24 (1.21 - 1.27)*** | 1.45 (1.42 - 1.48)*** |

Supplementary Table No 11: Numerical Data for Figure No. 1 and Figure No. 2

| Round of NFHS | Had knowledge without misconceptions | | Had knowledge with misconceptions | | No Knowledge | |
| --- | --- | --- | --- | --- | --- | --- |
|  | among men | among women | among men | among women | among men | among women |
| NFHS 3 | 21.01 | 16.74 | 34.09 | 33.39 | 44.9 | 49.88 |
| NFHS 4 | 34.18 | 27.57 | 37.63 | 41.58 | 28.19 | 30.85 |
| NFHS 5 | 27.7 | 23.37 | 42.69 | 46.46 | 29.6 | 30.17 |

Supplementary Table No 12: Numerical Data for Figure No. 3

| NFHS 3 Men | | NFHS 3 Women | | NFHS 3 PR | |
| --- | --- | --- | --- | --- | --- |
| State or Union Territory | No Knowledge | State or Union Territory | No Knowledge | State or Union Territory | No Knowledge |
| Andaman and Nicobar Islands | - | Andaman and Nicobar Islands | - | Andaman and Nicobar Islands | 0.00 |
| Andhra Pradesh | 42.66 | Andhra Pradesh | 39.66 | Andhra Pradesh | 1.08 |
| Arunachal Pradesh | 47.27 | Arunachal Pradesh | 54.57 | Arunachal Pradesh | 0.87 |
| Assam | 55.84 | Assam | 62.38 | Assam | 0.90 |
| Bihar | 53.85 | Bihar | 50.36 | Bihar | 1.07 |
| Chandigarh | - | Chandigarh | - | Chandigarh | 0.00 |
| Chhattisgarh | 59.73 | Chhattisgarh | 56.92 | Chhattisgarh | 1.05 |
| Dadra and Nagar Haveli | - | Dadra and Nagar Haveli | - | Dadra and Nagar Haveli | 0.00 |
| Daman and Diu | - | Daman and Diu | - | Daman and Diu | 0.00 |
| Goa | 53.33 | Goa | 43.15 | Goa | 1.24 |
| Gujarat | 42.29 | Gujarat | 44.71 | Gujarat | 0.95 |
| Haryana | 42.04 | Haryana | 54.43 | Haryana | 0.77 |
| Himachal Pradesh | 32.5 | Himachal Pradesh | 51.61 | Himachal Pradesh | 0.63 |
| Jammu and Kashmir | 59.46 | Jammu and Kashmir | 54.56 | Jammu and Kashmir | 1.09 |
| Jharkhand | 64.45 | Jharkhand | 60.33 | Jharkhand | 1.07 |
| Karnataka | 43.08 | Karnataka | 49.71 | Karnataka | 0.87 |
| Kerala | 36.17 | Kerala | 26.06 | Kerala | 1.39 |
| Ladakh | 59.46 | Ladakh | - | Ladakh | 0.00 |
| Lakshadweep | - | Lakshadweep | - | Lakshadweep | 0.00 |
| Madhya Pradesh | 44.08 | Madhya Pradesh | 37.51 | Madhya Pradesh | 1.18 |
| Maharashtra | 31.39 | Maharashtra | 30.73 | Maharashtra | 1.02 |
| Manipur | 21.94 | Manipur | 27.44 | Manipur | 0.80 |
| Meghalaya | 53.21 | Meghalaya | 48.59 | Meghalaya | 1.10 |
| Mizoram | 19.43 | Mizoram | 21.52 | Mizoram | 0.90 |
| Nagaland | 32.6 | Nagaland | 38.51 | Nagaland | 0.85 |
| Delhi | 20.12 | Delhi | 23.93 | Delhi | 0.84 |
| Odisha | 44.19 | Odisha | 57.41 | Odisha | 0.77 |
| Puducherry | - | Puducherry | - | Puducherry | 0.00 |
| Punjab | 52.56 | Punjab | 57.49 | Punjab | 0.91 |
| Rajasthan | 33.2 | Rajasthan | 53.69 | Rajasthan | 0.62 |
| Sikkim | 24.62 | Sikkim | 26.05 | Sikkim | 0.95 |
| Tamil Nadu | 24.86 | Tamil Nadu | 44.85 | Tamil Nadu | 0.55 |
| Telangana | 42.66 | Telangana | 39.66 | Telangana | 1.08 |
| Tripura | 59.17 | Tripura | 70.6 | Tripura | 0.84 |
| Uttar Pradesh | 41.01 | Uttar Pradesh | 44.94 | Uttar Pradesh | 0.91 |
| Uttarakhand | 38.02 | Uttarakhand | 47.93 | Uttarakhand | 0.79 |
| West Bengal | 53.6 | West Bengal | 56.92 | West Bengal | 0.94 |

Supplementary Table No 13: Numerical Data for Figure No. 4

| NFHS 4 Men | | NFHS 4 Women | | NFHS 4 PR | |
| --- | --- | --- | --- | --- | --- |
| State or Union Territory | No Knowledge | State or Union Territory | No Knowledge | State or Union Territory | No Knowledge |
| Andaman and Nicobar Islands | 17.57 | Andaman and Nicobar Islands | 16.2 | Andaman and Nicobar Islands | 1.08 |
| Andhra Pradesh | 26.03 | Andhra Pradesh | 26.74 | Andhra Pradesh | 0.97 |
| Arunachal Pradesh | 25.19 | Arunachal Pradesh | 39.87 | Arunachal Pradesh | 0.63 |
| Assam | 38.67 | Assam | 52.7 | Assam | 0.73 |
| Bihar | 34.4 | Bihar | 41.41 | Bihar | 0.83 |
| Chandigarh | 9.24 | Chandigarh | 21.62 | Chandigarh | 0.43 |
| Chhattisgarh | 25.68 | Chhattisgarh | 36.83 | Chhattisgarh | 0.70 |
| Dadra and Nagar Haveli | 27.48 | Dadra and Nagar Haveli | 37.77 | Dadra and Nagar Haveli | 0.73 |
| Daman and Diu | 27.48 | Daman and Diu | 41.46 | Daman and Diu | 0.66 |
| Goa | 3.47 | Goa | 19.08 | Goa | 0.18 |
| Gujarat | 30.71 | Gujarat | 39.53 | Gujarat | 0.78 |
| Haryana | 21.43 | Haryana | 17.68 | Haryana | 1.21 |
| Himachal Pradesh | 34.72 | Himachal Pradesh | 40.79 | Himachal Pradesh | 0.85 |
| Jammu and Kashmir | 29.74 | Jammu and Kashmir | 26.22 | Jammu and Kashmir | 1.13 |
| Jharkhand | 39.99 | Jharkhand | 52.5 | Jharkhand | 0.76 |
| Karnataka | 37.17 | Karnataka | 42.85 | Karnataka | 0.87 |
| Kerala | 22.97 | Kerala | 15.17 | Kerala | 1.51 |
| Ladakh | 29.74 | Ladakh | - | Ladakh | 1.13 |
| Lakshadweep | 39.06 | Lakshadweep | 37.93 | Lakshadweep | 1.03 |
| Madhya Pradesh | 34.8 | Madhya Pradesh | 33.37 | Madhya Pradesh | 1.04 |
| Maharashtra | 14.76 | Maharashtra | 19.64 | Maharashtra | 0.75 |
| Manipur | 15.43 | Manipur | 20.7 | Manipur | 0.75 |
| Meghalaya | 20.85 | Meghalaya | 26.95 | Meghalaya | 0.77 |
| Mizoram | 22 | Mizoram | 21.81 | Mizoram | 1.01 |
| Nagaland | 38.61 | Nagaland | 45.3 | Nagaland | 0.85 |
| Delhi | 15.67 | Delhi | 22.57 | Delhi | 0.69 |
| Odisha | 34.05 | Odisha | 36.34 | Odisha | 0.94 |
| Puducherry | 23.15 | Puducherry | 13.24 | Puducherry | 1.75 |
| Punjab | 19.01 | Punjab | 19.13 | Punjab | 0.99 |
| Rajasthan | 27.87 | Rajasthan | 34.11 | Rajasthan | 0.82 |
| Sikkim | 11.97 | Sikkim | 11.81 | Sikkim | 1.01 |
| Tamil Nadu | 24.25 | Tamil Nadu | 18.02 | Tamil Nadu | 1.35 |
| Telangana | 37.93 | Telangana | 28.66 | Telangana | 1.32 |
| Tripura | 49.68 | Tripura | 52.04 | Tripura | 0.95 |
| Uttar Pradesh | 31.22 | Uttar Pradesh | 26.99 | Uttar Pradesh | 1.16 |
| Uttarakhand | 27.12 | Uttarakhand | 36.29 | Uttarakhand | 0.75 |
| West Bengal | 37.72 | West Bengal | 43.69 | West Bengal | 0.86 |

Supplementary Table No 14: Numerical Data for Figure No. 5

| NFHS 5 Men | | NFHS 5 Women | | NFHS 5 PR | |
| --- | --- | --- | --- | --- | --- |
| State or Union Territory | No Knowledge | State or Union Territory | No Knowledge | State or Union Territory | No Knowledge |
| Andaman and Nicobar Islands | 16.06 | Andaman and Nicobar Islands | 23.65 | Andaman and Nicobar Islands | 0.68 |
| Andhra Pradesh | 26.41 | Andhra Pradesh | 26.53 | Andhra Pradesh | 1.00 |
| Arunachal Pradesh | 22.64 | Arunachal Pradesh | 26.98 | Arunachal Pradesh | 0.84 |
| Assam | 37.08 | Assam | 36.82 | Assam | 1.01 |
| Bihar | 40.91 | Bihar | 47.11 | Bihar | 0.87 |
| Chandigarh | 22.73 | Chandigarh | 25.27 | Chandigarh | 0.90 |
| Chhattisgarh | 24.56 | Chhattisgarh | 25.12 | Chhattisgarh | 0.98 |
| Dadra and Nagar Haveli | 42.16 | Dadra and Nagar Haveli | 22.02 | Dadra and Nagar Haveli | 1.91 |
| Daman and Diu |  | Daman and Diu |  | Daman and Diu |  |
| Goa | 7.38 | Goa | 5.89 | Goa | 1.25 |
| Gujarat | 30.8 | Gujarat | 35.02 | Gujarat | 0.88 |
| Haryana | 23.2 | Haryana | 24.81 | Haryana | 0.94 |
| Himachal Pradesh | 31.86 | Himachal Pradesh | 29.93 | Himachal Pradesh | 1.06 |
| Jammu and Kashmir | 21.82 | Jammu and Kashmir | 17.27 | Jammu and Kashmir | 1.26 |
| Jharkhand | 33.03 | Jharkhand | 51.22 | Jharkhand | 0.64 |
| Karnataka | 27.55 | Karnataka | 18.78 | Karnataka | 1.47 |
| Kerala | 12.44 | Kerala | 11.81 | Kerala | 1.05 |
| Ladakh | 15.28 | Ladakh | 16.68 | Ladakh | 0.92 |
| Lakshadweep | 10.92 | Lakshadweep | 13.51 | Lakshadweep | 0.81 |
| Madhya Pradesh | 29.62 | Madhya Pradesh | 30.4 | Madhya Pradesh | 0.97 |
| Maharashtra | 27.55 | Maharashtra | 22.68 | Maharashtra | 1.21 |
| Manipur | 12 | Manipur | 14.32 | Manipur | 0.84 |
| Meghalaya | 31.78 | Meghalaya | 31.72 | Meghalaya | 1.00 |
| Mizoram | 14.73 | Mizoram | 21.63 | Mizoram | 0.68 |
| Nagaland | 19.89 | Nagaland | 27.9 | Nagaland | 0.71 |
| Delhi | 19.96 | Delhi | 17.97 | Delhi | 1.11 |
| Odisha | 20.08 | Odisha | 25.67 | Odisha | 0.78 |
| Puducherry | 15.07 | Puducherry | 4.9 | Puducherry | 3.08 |
| Punjab | 31.55 | Punjab | 30.92 | Punjab | 1.02 |
| Rajasthan | 16.36 | Rajasthan | 21.19 | Rajasthan | 0.77 |
| Sikkim | 27.04 | Sikkim | 29.4 | Sikkim | 0.92 |
| Tamil Nadu | 17.8 | Tamil Nadu | 9.42 | Tamil Nadu | 1.89 |
| Telangana | 28.23 | Telangana | 22.57 | Telangana | 1.25 |
| Tripura | 45.08 | Tripura | 39.25 | Tripura | 1.15 |
| Uttar Pradesh | 29.95 | Uttar Pradesh | 33.88 | Uttar Pradesh | 0.88 |
| Uttarakhand | 33.43 | Uttarakhand | 25.5 | Uttarakhand | 1.31 |
| West Bengal | 45.86 | West Bengal | 49.57 | West Bengal | 0.93 |
